# Magnaporthe oryzae Auxiliary Activity Protein MoAa91 Functions as Chitin-Binding Protein To Induce Appressorium Formation on Artificial Inductive Surfaces and Suppress Plant Immunity

**DOI:** 10.1128/mBio.03304-19

**Published:** 2020-03-24

**Authors:** Ying Li, Xinyu Liu, Muxing Liu, Yang Wang, Yibin Zou, Yimei You, Lina Yang, Jiexiong Hu, Haifeng Zhang, Xiaobo Zheng, Ping Wang, Zhengguang Zhang

**Affiliations:** a Department of Plant Pathology, College of Plant Protection, Nanjing Agricultural University, Nanjing, China; b Key Laboratory of Integrated Management of Crop Diseases and Pests, Ministry of Education, Nanjing, China; c Department of Pediatrics, Louisiana State University Health Sciences Center New Orleans, New Orleans, Louisiana, USA; d Department of Microbiology, Immunology, and Parasitology, Louisiana State University Health Sciences Center New Orleans, New Orleans, Louisiana, USA; University of Texas Health Science Center

**Keywords:** *Magnaporthe oryzae*, auxiliary activity protein, appressorium, virulence, chitin binding

## Abstract

The appressoria that are generated by the rice blast fungus Magnaporthe oryzae in response to surface cues are important for successful colonization. Previous work showed that regulators of G-protein signaling (RGS) and RGS-like proteins play critical roles in appressorium formation. However, the mechanisms by which these proteins orchestrate surface recognition for appressorium induction remain unclear. Here, we performed comparative transcriptomic studies of Δ*Morgs* mutant and wild-type strains and found that M. oryzae Aa91 (MoAa91), a homolog of the auxiliary activity family 9 protein (Aa9), was required for surface recognition of M. oryzae. We found that *MoAA91* was regulated by the MoMsn2 transcription factor and that its disruption resulted in defects in both appressorium formation on the artificial inductive surface and full virulence of the pathogen. We further showed that MoAa91 was secreted into the apoplast space and was capable of competing with the immune receptor chitin elicitor-binding protein precursor (CEBiP) for chitin binding, thereby suppressing chitin-induced plant immune responses. In summary, we have found that MoAa91 is a novel signaling molecule regulated by RGS and RGS-like proteins and that MoAa91 not only governs appressorium development and virulence but also functions as an effector to suppress host immunity.

## Abstract (precis)

The rice blast fungus Magnaporthe oryzae generates infection structure appressoria in response to surface cues largely due to functions of signaling molecules, including G-proteins, regulators of G-protein signaling (RGS), mitogen-activated protein (MAP) kinase pathways, cAMP signaling, and TOR signaling pathways. M. oryzae encodes eight RGS and RGS-like proteins (MoRgs1 to MoRgs8), and MoRgs1, MoRgs3, MoRgs4, and MoRgs7 were found to be particularly important in appressorium development. To explore the mechanisms by which these proteins regulate appressorium development, we have performed a comparative *in planta* transcriptomic study and identified an auxiliary activity family 9 protein (Aa9) homolog that we named MoAa91. We showed that MoAa91 was secreted from appressoria and that the recombinant MoAa91 could compete with a chitin elicitor-binding protein precursor (CEBiP) for chitin binding, thereby suppressing chitin-induced plant immunity. By identifying MoAa91 as a novel signaling molecule functioning in appressorium development and an effector in suppressing host immunity, our studies revealed a novel mechanism by which RGS and RGS-like proteins regulate pathogen-host interactions.

## INTRODUCTION

The hemibiotrophic fungus Magnaporthe oryzae causes rice blast by producing an infectious structure called the appressorium that penetrates the host plant to initiate infection (1). This process requires the switch from polarized germ tube growth to appressorium formation upon perception of surface signals such as surface hardness and hydrophobicity. Other signals, such as cutin monomers (1,16-hexadecanediol) or components of plant epicuticular waxes (1-octacosanol), as well as environmental stimuli can also trigger appressorium formation (2–5).

In M. oryzae, G-protein/cAMP signaling plays an important role in the perception of host surface cues and the penetration of the host tissue (5, 6). M. oryzae contains three distinct Gα subunit proteins (M. oryzae MagA [MoMagA], MoMagB, and MoMagC) and a highly conserved cAMP-dependent signaling pathway, consisting of adenylate cyclase MoMac1 and cAMP-dependent protein kinase A CpkA/Cpk2 (7). Pth11, a noncanonical G-protein-coupled receptor (GPCR), was also found to sense signals and activate G-protein/cAMP signaling (8, 9). The mitogen-activated protein (MAP) kinase (MAPK) cascade, comprised of MoMst11 (MAPK kinase kinase [MAPKKK]), MoMst7 (MAPK kinase [MAPKK]), and MoPmk1 (MAPK), is also involved in transducing signals to regulate appressorium formation (10). MoPmk1 was shown to phosphorylate transcription factors that regulate the expression of gene involved in surface recognition. Moreover, two upstream sensors of the MAP kinase cascade, namely, surface mucin protein MoMsb2 and high osmolarity signaling protein MoSho1, were found to be important in appressorium formation (3). Moreover, TOR signaling was demonstrated to regulate appressorium formation via the cAMP/protein kinase A (PKA) pathway in M. oryzae (11, 12). We recently found that M. oryzae endocytic protein MoEnd3 mediates the internalization of Pth11 and MoSho1 to also regulate appressorium formation and virulence (13). All these studies indicated the complexity of the regulatory circuitry that governs virulence of the fungus.

For a successful infection, the pathogen not only generates functional appressoria but also needs to overcome rice immunity. Chitin, a microbe-associated molecular pattern (MAMP) in the apoplast of the host, can be detected by plant pattern recognition receptors (PRRs) that activate various MAMP-triggered immune responses (14). Pathogenic fungi often evolve to secrete effector proteins that either compete with the host receptors for chitin binding or reduce the accessibility of chitin to evade recognition by host receptors (15). In Cladosporium fulvum, effector Avr4 and extracellular protein Ecp6 bind to chitin, preventing its recognition by plant chitin receptors (16, 17). Similarly, in M. oryzae, secreted LysM domain protein MoSlp1 was shown to compete for chitin binding with the rice LysM–receptor-like protein (RLP) chitin elicitor-binding protein (CEBiP) that inhibits host immunity (18).

Regulators of G-protein signaling (RGS) are the negative regulators of G-protein signaling and perform the role through accelerating the GTP hydrolysis of Gα subunits, thereby rapidly switching off signal transduction (19, 20). Previously, we identified eight RGS and RGS-like proteins, MoRgs1 to MoRgs8, in M. oryzae and found that MoRgs1, MoRgs3, MoRgs4, and MoRgs7 were important in appressorium differentiation and virulence (21). However, it is still not clear how these RGS proteins mediate such functions. In an mRNA sequencing analysis (using transcriptome sequencing [RNA-Seq]) involving Δ*Morgs1*, Δ*Morgs3*, Δ*Morgs4*, and Δ*Morgs7* mutant strains, as well as wild-type Guy11, we identified three transcriptional patterns of genes regulated by RGS proteins during the early infection stage. In particular, we found a highly differentiated transcript of *MoAA91* that encodes a homolog of auxiliary activity family 9 (Aa9).

Aa9, originally identified from the common mushroom fungus *Agaricus bisporus* (22), catalyzes the cleavage of crystalline cellulose to improve the effectiveness of cellulase function (23, 24). In M. oryzae, the deletion of *MoAA91* resulted in delayed appressorium formation on artificial inductive surfaces and in attenuated virulence. These defects were rescued by a heterologously expressed MoAa91 protein. We also demonstrated that the enzymatic activity of MoAa91 was not required for its function in appressorium development and virulence. Moreover, MoAa91 shows high affinity in chitin binding and is capable of competing with CEBiP in binding chitin, thereby suppressing the chitin-induced plant immune response.

## RESULTS

### *MoAA91* is coregulated by four RGS genes based on RNA-Seq.

**FIG 1:**
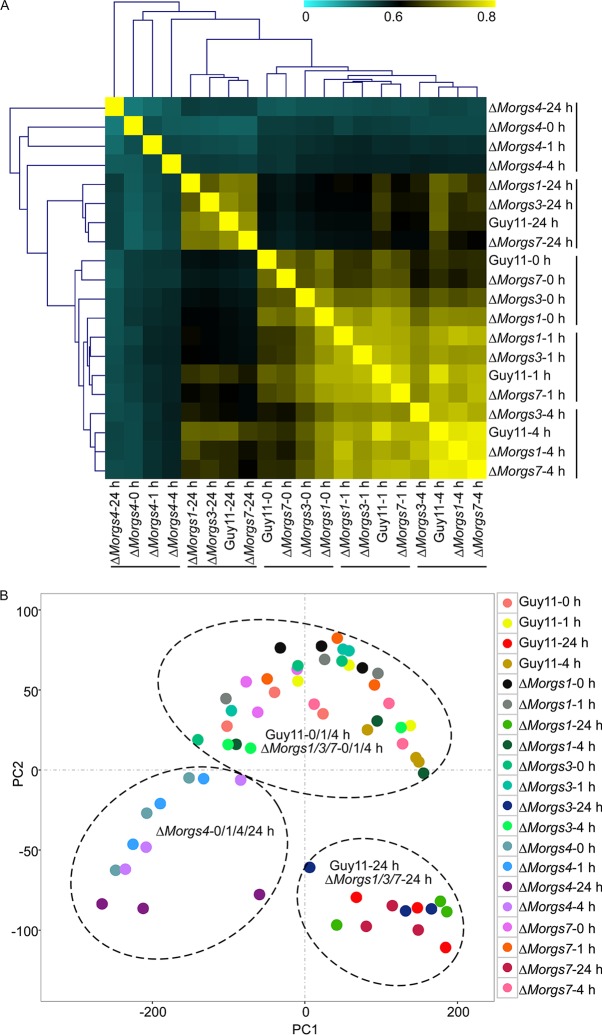
Expression dynamics of genes regulated by RGS proteins in M. oryzae. (A) Hierarchical cluster analysis was performed to compare gene expression levels in the Guy11, Δ*Morgs1*, Δ*Morgs3*, Δ*Morgs4*, and Δ*Morgs7* strains at 0, 1, 4, and 24 hpi. In general, the heat map indicates the pairwise distance between samples. The color bar represents the distances between the samples, with yellow signifying a small distance between the samples, while lighter blue signifies a larger distance. Gene expression levels in the Δ*Morgs4* mutant strain were different from those in the Δ*Morgs1*, Δ*Morgs3*, Δ*Morgs7*, and Guy11 strains at same time point postinfection. Gene expression levels at 24 hpi were different from those seen at the initial infected stages (0, 1, and 4 h). In general, the levels of gene expression of the Guy11, Δ*Morgs1*, Δ*Morgs3*, and Δ*Morgs7* strains exhibited less variability (yellow). However, the level of gene expression of the Δ*Morgs4* strain showed more variability (light blue). (B) Principal-component analysis (PCA) recapitulates the observation from the HCL analysis. The dotted circle at lower left indicates the level of gene expression of the Δ*Morgs4* mutant, the dotted circle at lower right indicates the gene expression of the Guy11, Δ*Morgs1*, Δ*Morgs3*, and Δ*Morgs7* strains at 24 hpi, and the upper part indicates expression of the Guy11, Δ*Morgs1*, Δ*Morgs3*, and Δ*Morgs7* strains at the initial infected stages (0, 1, and 4 hpi).

In M. oryzae, MoRgs1, MoRgs3, MoRgs4, and MoRgs7 are involved in appressorium development and virulence (21). To explore the molecular mechanism involved, we performed RNA-Seq analyses of the Guy11, Δ*Morgs1*, Δ*Morgs3*, Δ*Morgs4*, and Δ*Morgs7* strains inoculated on rice (CO39) at 0, 1, 4, and 24 h postinoculation (hpi). In total, we identified a total of 2,632 differentially expressed genes (DEGs), with 1,587 being upregulated and 1,045 downregulated, by comparing the Δ*Morgs* mutants to Guy11 at the same time points (see Fig. S1A in the supplemental material). To confirm the DEGs, the transcriptional patterns of 8 randomly selected DEGs were tested by quantitative real-time PCR (qRT-PCR). A total of 21 of 32 samples selected showed very similar fold change levels (Fig. S1B). In addition, hierarchical clustering (HCL) and principal-component analysis (PCA) were performed to assess the biological variability among all samples, and the results showed that the transcriptome of MoRgs4 was clearly different from those of other RGS genes (Fig. 1). The Venn analysis identified 36 genes that were coregulated by the four RGS proteins (Fig. S1C and D; see also Table S1A in the supplemental material), while most of the DEGs were independently regulated (Fig. S1C).

10.1128/mBio.03304-19.1 FIG S1 The differentially expressed genes and hierarchical cluster analysis. (A) The levels of expression of differentially expressed genes regulated by four *MoRGS* genes at the corresponding times were calculated and statistically analyzed. (B) Validation of detected genes by qRT-PCR. Eight genes of M. oryzae were randomly selected, and every two genes were regulated by four *MoRGS* genes at different infection stages and illuminated by the log_2_ RPKM value in different libraries. The fold change data represent the RPKM values calculated for different stages compared to the values seen with wild-type Guy11 at the corresponding times. Values for eight genes were calculated in the original sequenced samples by determination of the threshold cycle (*C_T_*) values from the results of qRT-PCR analysis compared to the values seen with wild-type Guy11 at the corresponding times. (C) Venn diagram of DEG clusters in different mutants. The numbers in the circles represent those of upregulated or downregulated genes at different infectious stages (0, 1, 4, and 24 hpi) by Δ*Morgs1*, Δ*Morgs3*, Δ*Morgs4*, and Δ*Morgs7* mutants compared to the results seen with wild-type Guy11. (D) Heat map of coregulated genes in four Δ*Morgs* mutants at different infectious stages. Gene fold change values determined for the Δ*Morgs1*, Δ*Morgs3*, Δ*Morgs4*, and Δ*Morgs7* mutants were compared separately against those determined for wild-type Guy11. Download FIG S1, TIF file, 1.9 MB. Copyright © 2020 Li et al. 2020 Li et al. This content is distributed under the terms of the Creative Commons Attribution 4.0 International license.

10.1128/mBio.03304-19.8 TABLE S1 (A) List of coregulated genes corresponding to the putative functions and the relative expression levels. (B) List of 215 differentially expressed secreted protein-encoding genes (DESGs) regulated by RGS proteins and their transcription profiles combined with the transcriptome of M. oryzae 98-06. (C) List of DESGs coregulated by four MoRgs proteins. (D) The homologous protein sequences of auxiliary activity family 9 in fungi. (E) List of transcriptional patterns of transcription factors combined with the transcriptome of M. oryzae 98-06. (F) Genes containing MoMsn2-binding sites in cluster A. (G) Primers used in this study. Download Table S1, XLSX file, 0.05 MB. Copyright © 2020 Li et al. 2020 Li et al. This content is distributed under the terms of the Creative Commons Attribution 4.0 International license.

**FIG 2:**
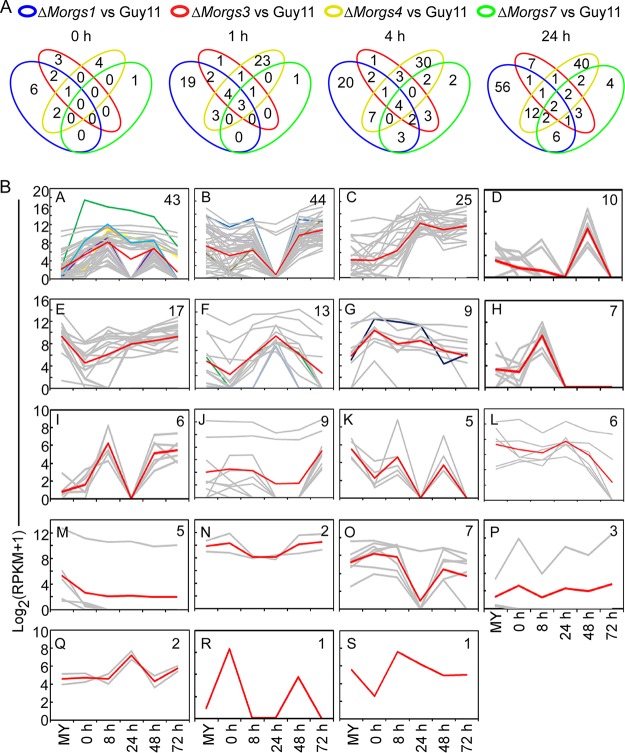
Transcription pattern characterization of DESGs in Δ*Morgs* mutants. (A) Venn analyses of the Δ*Morgs1*, Δ*Morgs3*, Δ*Morgs4*, and Δ*Morgs7* strains compared to Guy11 revealed that nine genes were shared in common. (B) Clustering analysis of gene expression patterns of DESGs combined with the transcriptome during the interaction between M. oryzae 98-06 and rice (26). The Pearson correlation coefficient calculated using an R script with comparisons between the levels of expression of these DESGs in the transcriptome of Guy11 and 98-06 at the appressorium stage was 0.81. The assay shows 19 M. oryzae expression clusters of DESGs. The *y*-axis data represent the log_2_ average gene expression levels. The quantity of cluster members is marked at the right top of each pattern line. The coregulated DESGs distributed into clusters A (5 genes), B (2 genes), F (1 gene), and G (1 gene). The red line refers to the average expression level, the gray refers to corresponding genes in the cluster, and the rest of the color lines refer to coregulated DESGs.

M. oryzae secretes various effectors to modulate host cellular processes, thereby suppressing host immunity and promoting infection (25). The total of 2,632 DEGs was narrowed to 215 genes that encode secreted proteins (Fig. 2A; see also Table S1B). Among them, 9 were found to be coregulated (Fig. 2A; see also Table S1C). We also observed a strong correlation (*R* = 0.81) between the expression levels of the differentially expressed secreted protein-encoding genes (DESGs) in Guy11 and 98-06 at the appressorium stage (Fig. S2A) in comparison to an earlier study published by us (26).

10.1128/mBio.03304-19.2 FIG S2 The Pearson correlation coefficient. (A) The Pearson correlation coefficient value calculated using an R script from comparisons between the levels of expression of the DESGs in the transcriptomes of strains Guy11 and 98-06 at the appressorium stage was 0.81. (B) The Pearson correlation coefficient value calculated from comparisons between the levels of expression of the transcription factors in the transcriptomes of Guy11 and 98-06 at the appressorium stage was 0.83 (the range of 0.8 to 1.0 refers to the relationships with the highest strength). Download FIG S2, TIF file, 0.2 MB. Copyright © 2020 Li et al. 2020 Li et al. This content is distributed under the terms of the Creative Commons Attribution 4.0 International license.

We further clustered the 215 DESGs based on the transcription pattern and identified 19 clusters (clusters A to S) (Fig. 2B; see also Table S2). Notably, five of the nine coregulated DESGs were grouped in cluster A, which consists of 20% DESGs. Since MGG_12939 (*MoCBP1*) and MGG_09055 (*MoCSR1*) were previously reported to play a crucial role in appressorium formation (27, 28), we generated knockout constructs for the remaining three (MGG_06069, MGG_02647, and MGG_16538) of those five genes and obtained the corresponding gene deletion mutants (Fig. S3A to D). Since only the MGG_06069 mutant exhibited apparent phenotypic changes whereas MGG_02647 did not (Fig. S3E to G), we thought to focus on characterizing its function. MGG_06069 is predicted to encode MoAa91 based on its high amino acid sequence identity to the auxiliary activity family 9 (Aa9) proteins (Fig. S4A; see also Table S1D) (24).

10.1128/mBio.03304-19.3 FIG S3 Southern blotting verified the mutants, and MGG_02647 was not involved in pathogenicity. (A) Construction strategy for knocking out of gene vector. Thick arrows indicate the orientations of the genes, including the *HPH* genes. Thin lines below the arrows indicate the probe sequence of each gene. (B to D) Southern hybridization was used to analyze disruption of the *MoAA91*, MGG_16538, and MGG_02647 genes. The restriction enzymes used for Southern blotting were EcoRI, HindIII, and XhoI, respectively. (E) Pathogenicity test of the MGG_02647 mutant grown on 2-week rice (cv. CO39) with incubation for 7 days. (F) Leaves from 3-week-old rice seedlings were injected with conidial suspension. Diseased leaves were photographed 7 dpi. (G) Pathogenicity test on barley leaves. Download FIG S3, TIF file, 1.2 MB. Copyright © 2020 Li et al. 2020 Li et al. This content is distributed under the terms of the Creative Commons Attribution 4.0 International license.

10.1128/mBio.03304-19.4 FIG S4 Secretion assay of auxiliary activity family 9 MoAa91 and transcriptional profiles of *MoAA91* and *MoMSN2* at different infectious stages. (A) Phylogenetic analysis of auxiliary activity family 9 in fungi. On the basis of protein sequences of glycoside hydrolase 61 domains, the phylogenetic tree was constructed using MEGA5.0 by the neighbor-joining method. The bootstrap replicates totaled 1,000. (B) Yeast invertase secretion assay of the predicted signal peptide of MoAa91. Yeast YTK12 strains carrying the MoAa91 signal peptide fragments fused in-frame to the invertase gene in the pSUC2 vector were able to grow in both CMD-W media (0.67% yeast N base without amino acids, 0.075% W dropout supplement, 2% sucrose, 0.1% glucose, and 2% agar) and YPRAA media (1% yeast extract, 2% peptone, 2% raffinose, and 2 mg/ml antimycin A) (with raffinose instead of sucrose; growth occurs only when invertase is secreted), as well as reduce 2, 3, 5-triphenyl tetrazolium chloride (TTC) to red formazan, indicating secretion of invertase. The controls included the untransformed YTK12 strain, YTK12 carrying the pSUC2 vector, and YTK12 carrying the pSUC2:PsAvr1b vector. (C) Microscopic observation of MoAa91:GFP fluorescence in appressorium formation on barley or rice sheath. (D) Transcriptional profiles of *MoAA91* and *MoMSN2* at different infectious stages. RNA was extracted from infectious stages (0, 2, 4, 6, 8, 10, 12, 16, 20, and 24 hpi with Guy11). The phase-specific expression of *MoAA91* and *MoMSN2* was quantified by qRT-PCR normalized to actin (MGG_03982), with the synthesis of cDNA from infectious growth. Three independent biological experiments were performed and yielded similar results. Error bars represent the standard deviations. Download FIG S4, TIF file, 1.2 MB. Copyright © 2020 Li et al. 2020 Li et al. This content is distributed under the terms of the Creative Commons Attribution 4.0 International license.

10.1128/mBio.03304-19.9 TABLE S2 List of transcriptional patterns of DESGs combined with transcriptome of M. oryzae 98-06. Download Table S2, XLSX file, 0.04 MB. Copyright © 2020 Li et al. 2020 Li et al. This content is distributed under the terms of the Creative Commons Attribution 4.0 International license.

### *MoAA91* is negatively regulated by the transcription factor MoMsn2.

**FIG 3:**
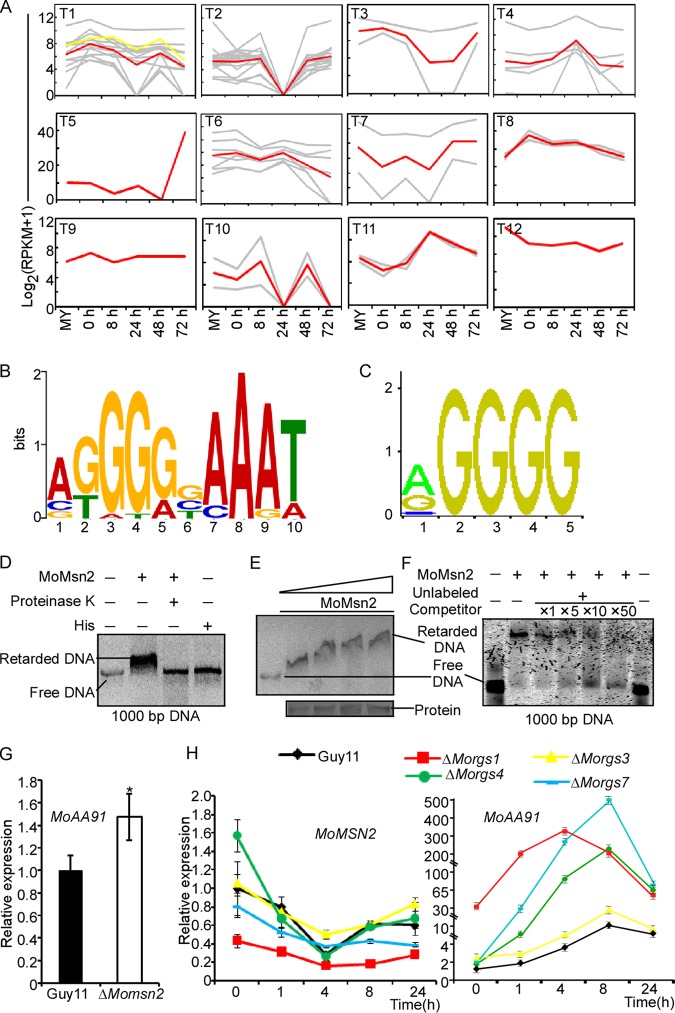
Genes in cluster A are regulated by MoMsn2. (A) Clustering analysis of gene expression patterns of differentially expressed transcription factors combined with the transcriptome of M. oryzae 98-06 (26). The assay shows 12 M. oryzae expression clusters of transcription factors. The *y*-axis data stand for the log_2_ average gene expression levels. The red line refers to the average expression level, the gray refers to corresponding genes in the cluster, and the yellow line refers to *MoMSN2*. (B) DNA motifs were generated by MEME from promoter analysis of the 43 genes in cluster A. (C) The putative binding motif of Msn2 in the JASPAR database. (D) The full-length DNA of the promoter of *MoAA91* was incubated in the absence (first lane) or presence (second lane) of purified MoMsn2 and in the presence of His protein (fourth lane). Proteinase K was added after the incubation of MoMsn2 with the DNA (third lane). DNA-protein complexes were separated by electrophoresis on a 1.5% agarose gel. (First lane) Full-length DNA of the promoter of *MoAA91*, as the control. (Second lane) The full-length DNA of the promoter of *MoAA91* was incubated in the presence of purified MoMsn2. (Third lane) Proteinase K was added after the incubation of MoMsn2 with the DNA to degrade the protein. (Fourth lane) The incubation of His protein with the DNA, as control. The results showed that the activity of the DNA was retarded by the addition of MoMsn2 in the second lane, suggesting that MoMsn2 protein was able to bind with the DNA, while proteinase K was able to dissolve the MoMsn2 protein and release DNA, and the His protein was not able to bind with DNA. (E) Increasing amounts of MoMsn2 were incubated with 1,000-bp DNA of the promoter of *MoAA91*. The complexes were resolved by electrophoresis on a 1.5% agarose gel. More MoMsn2 proteins bound to DNA, leading to the finding that the retardation of the DNA band was more significant. (F) MoMsn2 transcription factors were incubated with the Alex 660-labeled 1,000-bp DNA in the absence or presence of a 1×, 5×, 10×, or 50× excess of the corresponding unlabeled competitor DNA and analyzed by electrophoresis. We controlled the amount of MoMsn2 protein, and, with unlabeled competitor DNA levels increasing, the level of Alex 660-labeled 1,000-bp DNA bound with MoMsn2 protein became lower, indicating that Alex 660-labeled 1,000-bp DNA binding was weakened. (G) Expression of *MoAA91* in the Δ*Momsn2* and Guy11 strains. *MoAA91* was upregulated in the Δ*Momsn2* mutant. (H) Expression analysis of *MoAA91* (right) and *MoMSN2* (left) in the infected rice at 0, 1, and 4, 8, or 24 hpi with the wild-type Guy11 and Δ*Morgs1*, Δ*Morgs3*, Δ*Morgs4*, and Δ*Morgs7* mutant strains.

To study MoAa91 function, we first identified the transcription factors that are differentially expressed in the Δ*Morgs* mutants. A total of 57 transcription factors were differently expressed, and they were classified into 12 clusters (clusters T1 to T12) based on their expression levels. We found that the expression pattern of cluster T1 was similar to that of previously identified cluster A (Fig. 3A; see also Table S1E). Subsequently, the promoter sequences (600 bp) of cluster A were analyzed in MEME (http://meme-suite.org/tools/meme) and we found that 55.8% of the sequences shared a common motif (Fig. 3B; see also Table S1F). On searching the transcription factors of cluster T1 in the JASPAR database (http://jaspar.genereg.net), we identified a putative Msn2 binding site that shares the highest similarity with the motif predicted by MEME (Fig. 3C). MoMsn2 regulates a series of downstream genes that control appressorium formation and infection in M. oryzae (29), and we hypothesized that MoMsn2 regulates the cluster A genes.

Three cluster A genes (MGG_06494, MGG_07101, and MGG_09307) were randomly selected to verify the binding sequence with MoMsn2 using qRT-PCR and electrophoretic mobility shift assays (EMSAs) (Fig. S5). The combined results suggested that MoMsn2 indeed regulated the expression of the genes that possess the putative MoMsn2 binding motif within their promoter sequences. In addition, we verified that MoMsn2 showed binding to the promoter sequence of *MoAA91* by the use of EMSAs (Fig. 3D to F) and also found that the expression of *MoAA91* was upregulated in the Δ*Momsn2* mutant (Fig. 3G). We further analyzed the expression of *MoMSN2* and *MoAA91* during infection in the wild-type Guy11 strain and the Δ*Morgs* mutants and found that the two genes had opposing transcription profiles (Fig. 3H), suggesting that *MoAA91* is negatively regulated by MoMsn2.

10.1128/mBio.03304-19.5 FIG S5 MoMsn2 regulates the expression of MGG_06494, MGG_09377, and MGG_07101. (A) Expression levels of MGG_06494, MGG_07101, and MGG_09377 in the Δ*Momsn2* and Guy11 strains. (B) Promoter DNA of MGG_06494, MGG_07101, and MGG_09377 was incubated in the absence (first lane) or presence (second lane) of purified MoMsn2 and in the presence of His protein (fourth lane). Proteinase K was added after the incubation of MoMsn2 with the DNA (third lane). DNA-protein complexes were separated by electrophoresis on a 1.5% agarose gel. Download FIG S5, TIF file, 0.3 MB. Copyright © 2020 Li et al. 2020 Li et al. This content is distributed under the terms of the Creative Commons Attribution 4.0 International license.

### MoAa91 is important for pathogenicity.

**FIG 4:**
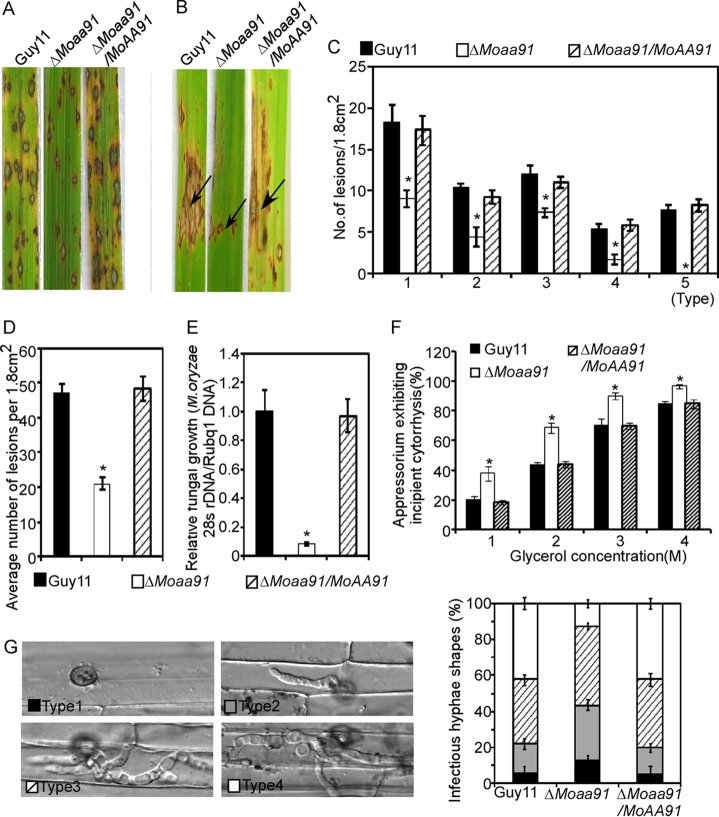
MoAa91 is critical for pathogenicity. (A) Disease symptoms on rice leaves were reduced by spraying of conidial suspensions inoculated with the Δ*Moaa91* mutant. The conidial suspensions (5 × 10^4^ spores/ml) of wild-type strain Guy11 and of the mutant and complemented strains were inoculated on rice (cv. CO39), and the rice was incubated for 7 days. Three independent experiments were performed. (B) Leaves from 3-week-old rice seedlings were injected with a conidial suspension (5 × 10^4^ spores/ml). Diseased leaves were photographed 7 days after inoculation. The arrows indicate the injection points. A total of 60 healthy rice seedlings were used for each strain in experiments performed three times. (C) Quantification of lesion types. Scoring was as follows: 0 = no lesion, 1 = pinhead-sized brown specks, 2 = 1.5-mm-diameter brown spots, 3 = 2-to-3-mm-diameter gray spots with brown margins, 4 = many elliptical gray spots longer than 3 mm, 5 = coalesced lesions infecting 50% or more of the leaf area. Lesions were photographed and measured or scored at 7 days postinoculation (dpi), and experiments were repeated three times with similar results. Numbers within an area of 1.8 cm^2^ of 20 rice leaves infected by each strain were counted. Asterisks indicate significant differences (Duncan’s new multiple-range tests, *P < *0.01). (D) Bar chart of mean lesion density of seedlings infected with wild-type strain Guy11 and the Δ*Moaa91* mutant per unit area. Mean lesion density was significantly reduced in the Δ*Moaa91* mutant infections. Error bars represent the standard deviations, and asterisks represent significant differences (Duncan’s new multiple-range tests, *P < *0.01). (E) The severity of blast disease was evaluated by quantifying M. oryzae genomic 28S ribosomal DNA (rDNA) relative to rice genomic Rubq1 DNA (7 dpi). The mean values of three determinations with standard deviations are shown. Asterisks represent significant differences (Duncan’s new multiple-range tests, *P < *0.01). (F) Appressorium turgor was measured by an incipient cytolysis (cell collapse) assay in the presence of 1, 2, 3, and 4 M glycerol. The percentage of collapsed appressoria was recorded by observing at least 100 appressoria, and the experiment was repeated three times. Error bars represent standard deviations, and asterisks represent significant differences (Duncan’s new multiple-range tests, *P < *0.01). (G) Penetration assays in rice sheath. IH growth on rice cells was observed at 30 hpi, and 4 types of IH were quantified and statistically analyzed (type 1, no penetration; type 2, a single primary hypha; type 3, extended but limited to one cell; type 4, spread to adjacent cells). Error bars represent standard deviations. A total of 50 invasive cells were statistically analyzed, and the experiment was repeated three times.

To examine the role of MoAa91 in the virulence of M. oryzae, conidial suspensions were sprayed onto susceptible rice (CO39) seedlings and injected into rice leaf sheaths. Seven days after inoculation, the Δ*Moaa91* mutant caused fewer and more-restricted lesions on the rice leaves (Fig. 4A and B). According to the “lesion type” scoring assay (30), most of the lesions caused by the Δ*Moaa91* mutant were of types 1 to 3, with very few being of type 4 and none of type 5 (Fig. 4C). The mean lesion density per unit area of the Δ*Moaa91* mutant was significantly lower than that of Guy11 (Fig. 4D). The level of fungal growth in rice was also significantly lower in the Δ*Moaa91* mutant (Fig. 4E). These results indicated that MoAa91 had a role in the full virulence of M. oryzae.

To understand the mechanism underlying the reduced virulence, the appressorium turgor was measured. We found that the collapse rates in the presence of 1, 2, 3, and 4 M glycerol were higher in the Δ*Moaa91* mutants (Fig. 4F), indicating that MoAa91 had influenced appressorium turgor generation. We also performed penetration assays using detached rice sheaths to assess the role of MoAa91 during the expansion of invasive hyphae (IH) in plant cells, using previously reported methods and criteria (31, 32). At 30 hpi, more than 40% of the IH of Guy11 and the complemented strains were type 4. In contrast, less than 12% of IH was type 4 in the Δ*Moaa91* mutant, while there were more types 1 to 3 seen with the Δ*Moaa91* mutant than with Guy11 and the complemented strains (Fig. 4G). These findings suggested that MoAa91 was important for penetration and invasive hyphal growth.

### MoAa91 is involved in regulating the normal appressorium formation on artificial inductive surfaces.

**FIG 5:**
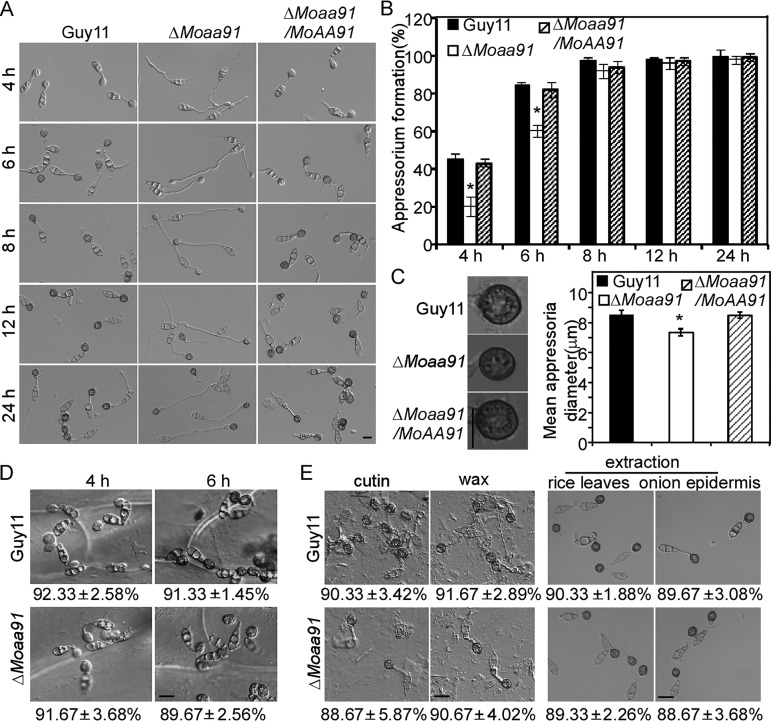
MoAa91 is involved in regulating appressorium formation on artificial inductive surfaces. (A) Appressorium formation assay. Conidia were incubated on artificial inductive surfaces, and the samples were observed at different time points. Bar = 10 μm. (B) Appressorium formation rates at different time points were calculated and statistically analyzed. The percentage at a given time was recorded by observing at least 100 conidia for each strain, and the experiment was repeated three times. Error bars represent standard deviations, and asterisks represent significant differences (Duncan’s new multiple-range tests, *P < *0.01). (C) (Left) Appressoria formed on artificial inductive surfaces after 24 h of incubation. (Bar = 10 μm.) (Right) The mean appressorium diameter was recorded by measuring at least 100 appressoria for each strain, and the experiment was repeated three times. Error bars represent standard deviations, and the asterisk represents a significant difference (Duncan’s new multiple-range tests, *P < *0.01). (D) Conidia of the wild-type Guy11 and Δ*Moaa91* mutant strains were inoculated onto onion epidermis in 20-μl droplets followed by incubation for 4 or 6 h. (E) Appressorium development was observed in the wild-type Guy11 and Δ*Moaa91* mutant strains in the presence of cutin (1,16-hexadecanediol) and wax (1-octacosanol) followed by extraction of rice leaves and onion epidermis for 24 h, respectively. Bar = 10 μm.

By observing appressorium formation on artificial inductive surfaces, we found that the Δ*Moaa91* mutant had a delay in appressorium differentiation compared with the wild-type Guy11 strain and the complemented strain. The percentage of appressorium formation was significantly lower in the Δ*Moaa91* mutant at 4 and 6 hpi, but it became less distinguishable after 8 hpi (Fig. 5A and B). However, the germ tubes of the Δ*Moaa91* mutant were longer, and the appressoria were also smaller and not fully developed until 24 hpi (Fig. 5A and C). These results suggested that MoAa91 contributed to appressorium development on the artificial inductive surface.

Surprisingly, we found that the Δ*Moaa91* mutant formed normal germ tubes and appressoria on onion epidermis, which was different from the results seen with an artificial inductive surface (Fig. 5D). Given that the artificial inductive surface lacked the signaling molecules from the rice leaf surface, such as the cutin monomer (1,16-hexadecanediol) or wax (1-octacosanol), we speculated that MoAa91 played a role in the recognition of surface signals. To test this hypothesis, we examined appressorium formation of the Δ*Moaa91* mutant on an artificial inductive surface coated with cutin and wax or in the presence of rice and onion epidermis extracts and found that the Δ*Moaa91* mutant produced normal germ tubes and appressoria at 24 hpi under all of these conditions (Fig. 5E). These results suggested that the deletion of *MoAA91* did not impair the ability of the fungus to sense plant surface signals during appressorium formation.

### MoAa91 is secreted during appressorium development and is synergistic with cellulase.

**FIG 6:**
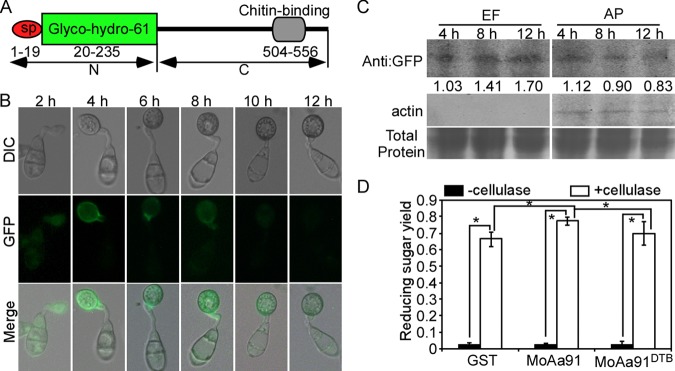
MoAa91 is secreted at the appressorium development and is synergistic with cellulase. (A) Diagram of MoAa91. The red oval refers to signal peptide, the green box refers to the Glyco_hydro_61 domain, and the rounded gray box refers to the chitin-binding domain (504 to 556). N, N-terminal sequence of amino acids 20 to 235 (named MoAa91^N^); C, C-terminal sequence of amino acids 236 to 603 (named MoAa91^C^). (B) Microscopic observation of the MoAa91-GFP fluorescence in appressorium formation on the artificial inductive surface. DIC, differential interference contrast. (C) Western blot analysis of proteins in the extracellular fluid (EF) of the appressorium (AP) at 4, 8, and12 hpi on inductive surfaces and corresponding appressoria of the complemented strain expressing MoAa91:GFP. The lower panel shows total proteins by Coomassie blue staining. The middle panel shows actin specific to M. oryzae. Proteins of extracellular fluid did not contain the component of M. oryzae. The appressorium proteins were present in the same amounts. Each number corresponds to the gray area shown in the upper panel, indicating that MoAa91:GFP was secreted from the appressorium to extracellular fluid. (D) Enzyme activity of MoAa91 on Avicel PH-101 substrates and synergistic activities of MoAa91 and MoAa91^DTB^ in the hydrolysis of cellulose by Celluclast. Error bars represent standard deviations, and the asterisk represents a significant difference (Duncan’s new multiple-range tests, *P < *0.01).

To explore the mechanism underlying the action of MoAa91 in appressorium formation and pathogenicity, we first analyzed its structure and found that MoAa91 contains a signal peptide, a Glyco_hydro_61 domain, and a C-terminal chitin-binding domain (Fig. 6A). The signal peptide was validated (Fig. S4B) based on a method previously described by Oh et al. (33). We then investigated whether MoAa91 was expressed and secreted during appressorium formation. MoAa91 was highly expressed at 4 hpi, and the expression weakened gradually to become nearly nondetectable at 12 hpi on the artificial inductive surface (Fig. 6B). Similar results were observed on barley leaves and rice sheaths (Fig. S4C). However, the level of *MoAA91* transcription during appressorium formation reached a higher value at 8 hpi, and the expression of *MoAA91* increased from 4 to 8 hpi, while the green fluorescent protein (GFP) fluorescence weakened gradually (Fig. S4D). We postulated that MoAa91 was secreted during this period. Therefore, we examined the expression of MoAa91 in the extracellular fluid (EF) collected from the complemented strain during appressorium formation on an artificial inductive surface by Western blotting. We found that the level of MoAa91 increased in EF and decreased in appressoria from 4 to 12 hpi (Fig. 6C), consistent with the idea that MoAa91 is secreted from the appressoria.

We also assessed the MoAa91 enzyme activity and found that MoAa91 alone showed no hydrolase activity but showed synergistic activity when incubated with the cellulase (Fig. 6D). On the basis of a previous study (34), we predicted that residues 20, 102 (histidines), and 186 (tyrosine) formed the active sites of MoAa91 (Fig. S6) and we therefore constructed a point mutation of MoAa91, namely, MoAa91^DTB^ (H20A/H102A/T186A), and analyzed its enzyme activity. The result revealed that these residues were required for the synergistic activity of MoAa91 with the cellulase (Fig. 6D). But we determined that this synergistic activity is not necessary for the function of appressorium formation and virulence on the basis of the experiments described below.

10.1128/mBio.03304-19.6 FIG S6 Active-site prediction of MoAa91. (A) Active-site prediction of MoAa91 based on Li’s method (34). Copper ion is shown as a brown sphere. (B) Structure-based sequence alignment of MoAa91 with known X-ray crystal structures of Neurospora crassa particulate methane monooxygenases (PMOs). Conserved residues are shaded in gray, and asterisks mark the conserved active sites. Download FIG S6, TIF file, 1.1 MB. Copyright © 2020 Li et al. 2020 Li et al. This content is distributed under the terms of the Creative Commons Attribution 4.0 International license.

### MoAa91 acts as an inducer to regulate appressorium differentiation.

**FIG 7:**
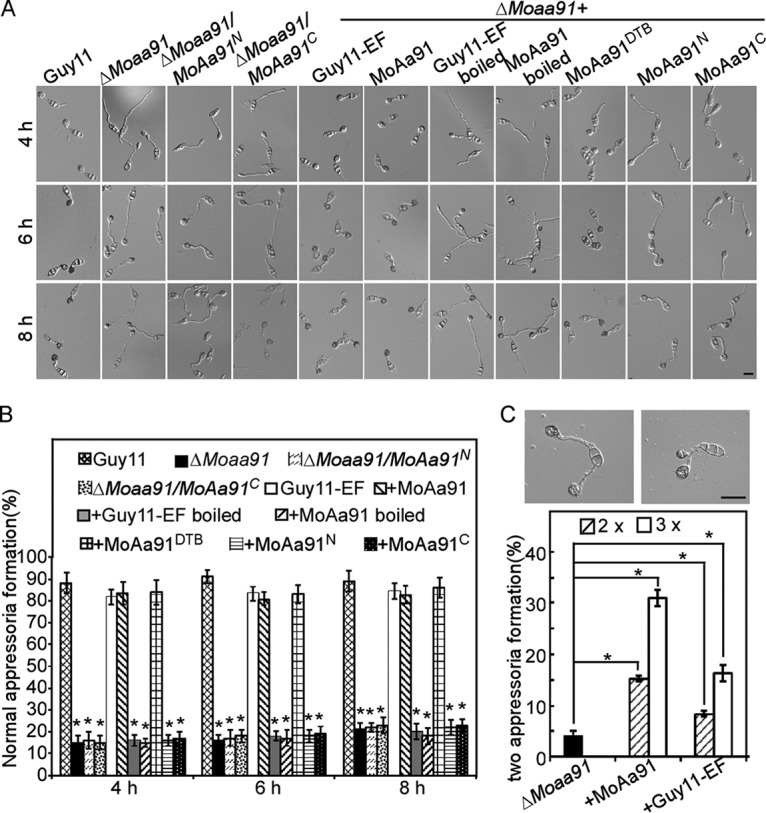
MoAa91 acts as an inducer to regulate the development of appressoria on the artificial inductive surface. (A) Guy11-EF and purified expressed MoAa91 or MoAa91^DTB^ in Escherichia coli were added into the conidial suspension of the Δ*Moaa91* mutant and suppressed the appressorium defects of mutants on inductive surfaces. However, the appressoria were abnormal after treatment with boiled Guy11-EF. The Δ*Moaa91*/*MoAA91*^N^-GFP and Δ*Moaa91*/*MoAA91*^C^-GFP transformants showed defects in appressorium differentiation similar to that shown by the Δ*Moaa91* mutant, as well as to that seen after addition of heterologously expressed MoAa91^N^ or MoAa91^C^ proteins to the conidial suspension. Apart from the Guy11 control (first column), columns 5 to 11 represent proteins added into the conidial suspension of the Δ*Moaa91* mutant. (B) Normal appressorium formation rates at different time points were calculated and statistically analyzed. (The appressorium formed by the Δ*Moaa91* mutant had long germ tubes, while the appressorium formed by the wild-type strain had short germ tubes.) The percentage present at a given time was recorded by measuring at least 100 conidia for each treatment, and the experiment was repeated three times. Error bars represent standard deviations, and asterisks represent significant differences (Duncan’s new multiple-range tests, *P < *0.01). (C) Two appressoria were formed at the tip of one germ tube treated with 2× or 3× Guy11-EF and purified MoAa91. Formation rates of two appressoria were calculated and statistically analyzed. “2 x” or “3 x” refers to 2× or 3× Guy11-EF and purified MoAa91, respectively, amounts which can restore normal appressorium formation. The percentage at a given concentration was recorded by observing at least 100 conidia for each treatment, and the experiment was repeated three times. Error bars represent standard deviations, and asterisks represent significant differences (Duncan’s new multiple-range test, *P < *0.01).

MoAa91 is not involved in the recognition of physical or chemical stimuli during appressorium differentiation (Fig. 5D and E). To explore how the deletion of *MoAA91* could cause defects in appressorium differentiation on artificial inductive surfaces, appressorium formation was examined by inoculating Δ*Moaa91* conidia mixed with EF on an artificial inductive surface. After 4 hpi, the conidia of the Δ*Moaa91* mutant formed normal germ tubes and appressoria (Fig. 7A and B), indicating that some components of the Guy11-EF rescued appressorium formation by the Δ*Moaa91* mutant, whereas boiled Guy11-EF failed to suppress this defect (Fig. 7A and B). Interestingly, adding heterologously expressed MoAa91 to the conidial suspension on an artificial inductive surface suppressed the appressorium formation defects of the Δ*Moaa91* mutant. Moreover, increasing the concentration of Guy11-EF or MoAa91 protein enhanced the ratio of two appressoria formed from one conidium (Fig. 7C). Surprisingly, adding the nonactive MoAa91 protein (MoAa91^DTB^) also suppressed the defects of the mutant (Fig. 7A), indicating that the enzyme activity of MoAa91 is not required for appressorium differentiation. In addition, to further assess the role of the motifs, we divided MoAa91 into two parts, the N-terminal domain (i.e., the Glyco_hydro_61 domain; indicated as “N” in Fig. 6A) and the C-terminal domain (indicated as “C” in Fig. 6A), and obtained the Δ*Moaa91*/*MoAA91*^N^-GFP and Δ*Moaa91*/*MoAA91*^C^-GFP transformants. We found that the defect in the appressorium differentiation of these two types of transformants was similar to that of the Δ*Moaa91* mutant, as well as to that seen after adding purified MoAa91^N^ or MoAa91^C^ proteins expressed in Escherichia coli to the conidial suspension (Fig. 7A and B). On the basis of these results, we speculated that MoAa91 was secreted into extracellular space and that the two motifs of MoAa91 were essential for the functions of the appressorium inducer that regulated the appressorium formation on artificial inductive surfaces.

### MoAa91 is involved in suppressing host defense.

**FIG 8:**
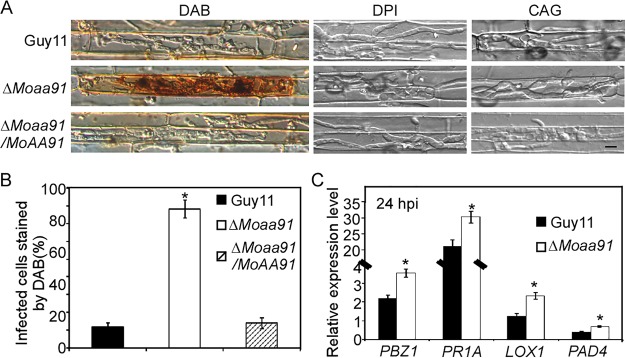
MoAa91 was involved in suppressing host defense. (A) 3,3′-Diaminobenzidine (DAB) staining of the excised leaf sheath of rice infected by Guy11, the Δ*Moaa91* mutant, and the complemented strain at 30 hpi. The excised sheath of rice was inoculated with the conidial suspension after treatment with 0.5 μM diphenyleneiodonium (DPI) dissolved in dimethyl sulfoxide (DMSO) or with 0.2 U catalase of Aspergillus niger (CAG; Sigma) dissolved in 10 mM (NH4)_2_SO_4_ in three independent experiments. Samples were harvested and were observed 30 hpi. This experiment was performed independently three times, and representative results from one of these experiments are presented. (Bar = 10 μm.) (B) The infected cell was stained by DAB. Three independent biological experiments were performed with three replicates each time, yielding similar results in all of the independent biological experiments. Error bars represent standard deviations; asterisks represent significant differences between the different strains (*P* < 0.01) based on Duncan’s new multiple-range tests. (C) The transcription of *PBZ1*, *PR1A*, *LOX1*, and *PAD4* in the infected rice was assayed using qRT-PCR. RNA samples were collected from rice leaves 24 hpi with wild-type strain or the Δ*Moaa91* mutant. The average threshold cycle values of triplicate reactions were normalized to the stable expression levels of gene elongation factor 1 (Os03G08020) in Oryza sativa. Three independent biological experiments were performed and yielded similar results. Error bars in the figure represent standard deviations; asterisks denote statistical significance (*P < *0.01) based on Student's *t* test.

Reactive oxygen species (ROS) function as secondary signals that mediate plant defense (35–38). As the Δ*Moaa91* mutant showed restricted growth of infectious hyphae, we hypothesized that MoAa91 was involved in suppressing the host defense. To test this, host-derived ROS was detected by staining with 3,3′-diaminobenzidine (DAB) at 30 hpi of the rice cells infected with the Δ*Moaa91* mutant; we found that 82% of the infected cells stained brown, compared with 12% and 15% of those infected with the wild-type and complemented strains, respectively. This finding indicated that the Δ*Moaa91* mutant failed to suppress ROS surrounding the site of infection (Fig. 8A).

Further, we employed diphenyleneiodonium (DPI), an inhibitor of the activity of NADPH oxidases, which is necessary for ROS generation in plants (39–42). Upon treatment with 0.5 μM DPI, the infectious hyphae of the Δ*Moaa91* mutant spread to the neighboring cells (Fig. 8A). Treatment with catalase (CAG; Sigma), which degrades H_2_O_2_ surrounding the infection site, suppressed the infection defect of the Δ*Moaa91* mutants (Fig. 8A and B). These results demonstrated that inhibiting ROS generation or scavenging ROS suppressed the infection defects of the Δ*Moaa91* mutants.

We then examined the transcript levels of pathogenesis-related (PR) genes, including *PBZ1*, *LOX1*, *PR1A*, and *PAD4*, which are involved in the jasmonic acid (JA) pathway or the salicylic acid (SA) pathway (26, 42). The transcript levels of these PR genes were higher in rice leaves infected with the Δ*Moaa91* mutant than in those infected with the wild-type Guy11 strain (Fig. 8C). On the basis of these results, we concluded that restriction of the Δ*Moaa91* mutant in rice cells might be due to host-derived ROS accumulation at the infection site and the subsequent activation of a strong host defense response.

### MoAa91 is a competitive inhibitor of rice receptor CEBiP that suppresses chitin-triggered plant immune responses.

**FIG 9:**
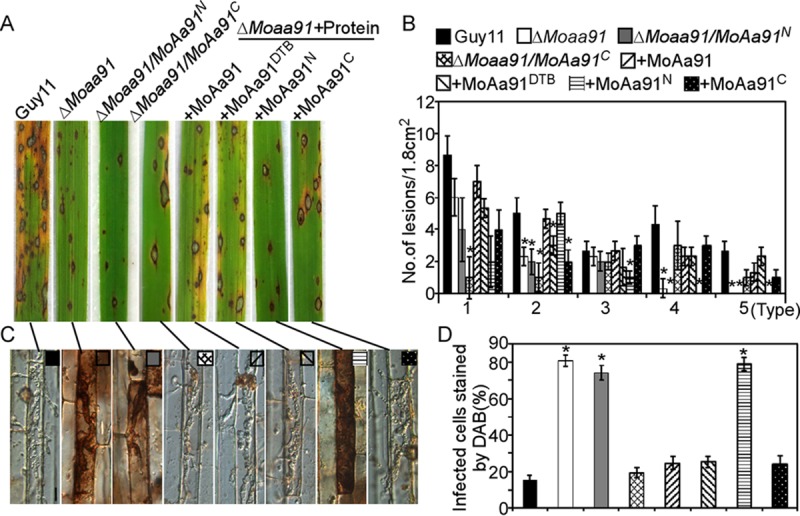
The chitin-binding domain was responsible for the pathogenicity of MoAa91. (A) Levels of disease symptoms were increased on rice leaves inoculated with a Δ*Moaa91*/*MoAA91*^C^-GFP mutant and Δ*Moaa91* mutant in a mixture with heterologously expressed MoAa91, MoAa91^DTB^, or MoAa91^C^ compared with Δ*Moaa91* mutant results. The conidial suspension (5 × 10^4^ spores/ml) of wild-type strain Guy11, the Δ*Moaa91* mutant, Δ*Moaa91*/*MoAA91*^N^-GFP and Δ*Moaa91*/*MoAA91*^C^-GFP mutants, and mutants mixed with heterologously expressed strain MoAa91, MoAa91^DTB^, MoAa91^N^, or MoAa91^C^ was sprayed to inoculate 2-week-old rice seedling (cv. CO39), and the seedlings were incubated for 7 days. (B) Lesions were photographed and measured or scored at 7 dpi, and experiments were repeated three times with similar results. Numbers within an area of 1.8 cm^2^ were counted. Asterisks indicate significant differences (Duncan’s new multiple-range tests, *P < *0.01). (C) 3,3′-Diaminobenzidine (DAB) staining of the excised leaf sheath of rice infected by Guy11, the Δ*Moaa91* mutant, Δ*Moaa91*/*MoAA91*^N^-GFP and Δ*Moaa91*/*MoAA91*^C^-GFP mutants, and mutants mixed with heterologously expressed strain MoAa91, MoAa91^DTB^, MoAa91^N^, or MoAa91^C^ 30 hpi. (Bar = 10 μm.) (D) The infected cell stained by DAB. Three independent biological experiments were performed, with three replicates each time, yielding similar results in all independent biological experiments. Error bars represent standard deviations; asterisks represent significant differences (Duncan’s new multiple-range tests, *P < *0.01).

Given that MoAa91 is a secreted protein and that deletion of the *MoAA91* gene leads to the accumulation of ROS in infected cells, we postulated that MoAa91 acted as an effector once secreted into the host cell or the apoplastic space to suppress plant immunity. Therefore, we added the respective recombinant MoAa91 and MoAa91^DTB^ proteins to the conidial suspension of the Δ*Moaa91* mutant and sprayed the suspension onto rice seedlings. The disease symptoms were severe, with more type 4 and 5 lesions produced than were seen with the Δ*Moaa91* mutant alone (Fig. 9A and B). We then examined the ROS surrounding the infected site and found that the percentages of cells stained by DAB in the Δ*Moaa91* mutant expressing MoAa91 and MoAa91^DTB^ were similar to those seen with the wild-type strain (Fig. 9C and D). These results suggested that heterologously expressed MoAa91 or MoAa91^DTB^ was able to suppress the defense response of rice and facilitate the growth of the invasive hyphae.

Furthermore, we dissected the function of MoAa91 by separating it into the N-terminal glycoside hydrolase 61 domain and the C-terminal chitin-binding domain. We found that the Δ*Moaa91*/*MoAA91*^C^-GFP transformants suppressed the defect in pathogenicity of the Δ*Moaa91* mutant (Fig. 9A). This result was in agreement with the finding that adding purified MoAa91^C^ proteins to the conidial suspension rescued the defects of Δ*Moaa91* mutant (Fig. 9C and D). On the basis of these results, it is suggested that the C-terminal domain was important in the pathogenicity of MoAa91.

**FIG 10:**
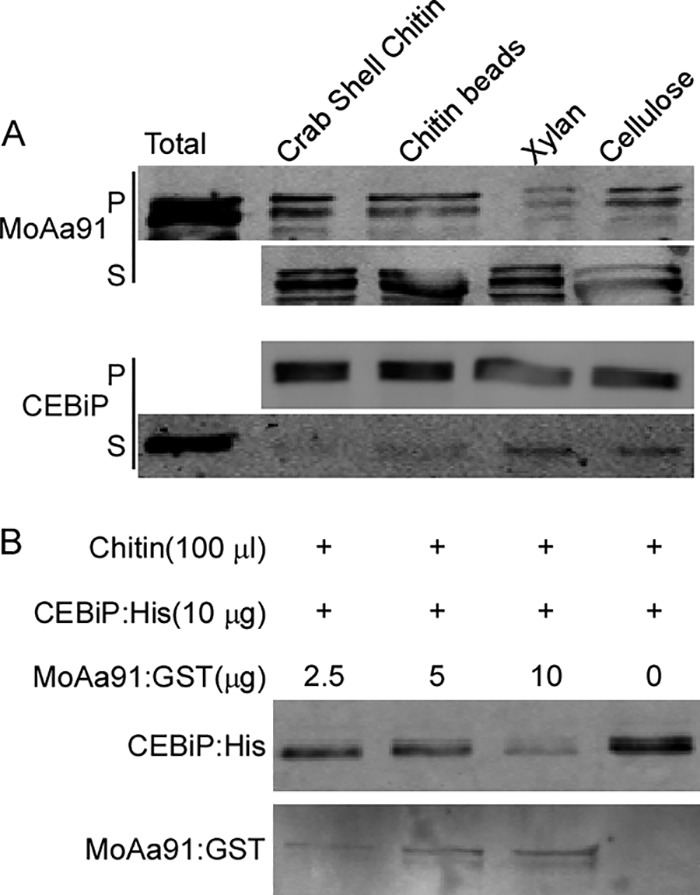
MoAa91 binds to chitin oligosaccharides, and MoAa91 is a competitive inhibitor of the rice CEBiP receptor. (A) Affinity precipitation experiments showing that MoAa91 or CEBiP coprecipitates with insoluble crab shell chitin and chitin beads and that the results were detected in the insoluble pellet fraction (P) and the soluble supernatant fraction (S) following SDS-PAGE and Western blotting using anti-GST or anti-His. MoAa91 showed thinner precipitate with insoluble polysaccharides, including xylan and cellulose (Avicel PH-101), than with others. (B) Western blot analysis using an anti-His or anti-GST antibody, showing affinity labeling of the PRR CEBiP, with chitin beads, in the presence or absence of MoAa91. The experiment was performed twice with similar results.

As MoAa91^C^ contains the chitin-binding domain, we hypothesized that MoAa91 was capable of binding specific polysaccharides, including chitin. After incubation of the purified MoAa91 protein with insoluble chitin (chitin beads and crab shell chitin), xylan polysaccharides, and cellulose (Avicel), we observed that MoAa91 coprecipitated with insoluble chitin at higher levels than with polysaccharides (Fig. 10A). As previous studies had demonstrated that rice PRR CEBiP directly bound chitin oligosaccharides, leading to subsequent strong activation of the plant defense response (43), we purified CEBiP:His with insoluble chitins (Fig. 10A). Therefore, we hypothesized that MoAa91 might be involved in disrupting chitin-induced sensing mediated by CEBiP between the fungus and the rice host. To test whether MoAa91 competes with CEBiP for chitin binding, a competition assay was performed by incubation of the CEBiP:His protein (10 μg) with chitin beads (100 μl) followed by the addition of MoAa91 (Fig. 10B). The results showed that the level of CEBiP-bonded chitin became significantly reduced as the concentration of MoAa91 increased, suggesting that MoAa91 indeed competes with CEBiP in chitin binding.

## DISCUSSION

RNA-Seq analysis revealed that MoRgs1, MoRgs3, MoRgs4, and MoRgs7 regulated the expression of various genes during the early infection stages and that the regulation levels appeared to be distinct and to vary with the different RGS proteins. Of note, there were significant differences in the genes regulated by MoRgs4. On analyzing the transcription pattern of 215 DESGs, 19 different expression patterns were found, including patterns corresponding to cluster A, cluster B, and cluster C. Cluster A contains five genes coregulated by the aforementioned four MoRgs, including MoAa91, which was shown to be a protein that is secreted during appressorium formation. While the receptor(s) specific to MoAa91 is not known, we did find that MoAa91 functioned as an inducer of appressorium formation on artificial inductive surfaces. Despite the finding that disruption of *MoAA91* did not impair the ability of M. oryzae to sense the plant surface cue, evidence was accumulated to demonstrate that MoAa91 is involved in suppressing the host-derived defense response to promote infection.

We found that most of the DESGs showed one of the three expression patterns, i.e., the cluster A, cluster B, or cluster C expression pattern. Cluster B showed the expression pattern of pathogenicity-related genes, and cluster C showed the expression pattern of effector genes, including *BAS1*, *BAS3*, *BAS4*, and *AvrPiz-t* (26). From these two clusters, several novel pathogenicity-related genes and effector genes were identified, such as *IUG18*, *IUG6*, and *IUG9* (26). Only cluster A, which showed an “M”-like expression pattern, has not been reported previously. Here, we identified five genes that were coregulated by four MoRgs proteins with the cluster A pattern. Other genes with a similar expression pattern, such as those encoding MoCbp1 and MoCsr1, are also involved in appressorium development (27, 28). Thus, the cluster A expression pattern appears to be important for identifying novel genes and effector genes related to appressorium formation. Further study showed that various genes in the cluster A were regulated by transcription factor MoMsn2, suggesting that MoMsn2 may be the key regulator of MoRgs-mediated signaling during appressorium formation in M. oryzae.

In M. oryzae, conidia germinate to form germ tubes that further differentiate into appressoria through sensing surface hardness, hydrophobicity, and additional physical or chemical cues (44). Multiple putative sensors that respond to physical and chemical cues on rice leaves have been reported previously (45). Pth11 is involved in recognizing surface cues on rice leaves and is required for appressorium formation (8). The surface mucin protein MoMsb2 and extracellular chitin-binding protein MoCbp1 are important for appressorium formation on artificial inductive surfaces (27). MoMsb2 is also critical for sensing hydrophobicity and cutin monomers on the rice surface (3). Studies also identified the conserved fungal-specific extracellular membrane-spanning (CFEM) GPCR WISH that was required for appressorium differentiation on an inductive surface or rice leaf. WISH has a critical role in the recognition of hydrophobicity and appressorium morphogenesis (46). In the current study, MoAa91 did not seem to have a role in sensing plant surface signals as the ability of the Δ*Moaa91* mutant to form appressoria on leaf surfaces was not affected, even though it was delayed on artificial surfaces.

It was reported previously that the chitin-deacetylase activity of MoCbp1 plays a vital role in the induction phase of appressorium formation (47). MoAa91 showed synergistic activity with cellulase, but such synergy remained minor, similarly the levels reported in a previous study of other Aa9 protein homologs (34). In addition, both MoAa91 and MoAa91^DTB^ were able to overcome the defect in appressorium formation on artificial surfaces. We therefore speculated that M. oryzae perceived the structure of MoAa91 protein, rather than the enzymatic activity, in regulating appressorium development. On plant surfaces, such as rice leaves, despite MoAa91 still being secreted, the fungus may preferentially perceive stimuli from cutin and waxes.

Pathogens secrete various effectors that are hypothesized to facilitate effective host infection (48). Some effectors are secreted by appressoria even before the penetration of the host (49). A Colletotrichum higginsianum effector candidate (ChEC) was expressed in appressoria only before penetration and might have been secreted through a pore in melanized appressoria (50–52). The unique M. oryzae *AVR* gene *ACE1* encodes avirulence-conferring enzyme 1, which is localized specifically to the cytoplasm of appressoria. An uncharacterized secondary metabolite synthesized by the AceI enzyme is secreted from the appressorium, triggering *Pi33*-mediated resistance in rice (53–55). Our results revealed that the level of expression of *MoAA91* was increased in the appressorium stage and that MoAa91 was secreted during appressorium development. Interestingly, heterologously expressed MoAa91 or MoAa91^DTB^ was able to suppress the ROS and partially restore the pathogenicity defect. Therefore, it is reasonable to postulate that MoAa91 is an effector involved in disrupting host-derived immunity. It is not clear how MoAa91 is secreted into the host cells or into the apoplastic spaces of host cells. Previous studies showed that there are two independent secretion pathways involved in effector transport into or around the host cells: the biotrophic interfacial complex (BIC) pathway and the extrainvasive hyphal membrane (EIHM) pathway (49). However, no fluorescence of GFP-MoAa91 was observed in rice cells during infection. Therefore, we speculated that MoAa91 was likely secreted through an appressorium pore similar to that seen with ChEC of C. higginsianum.

**FIG 11:**
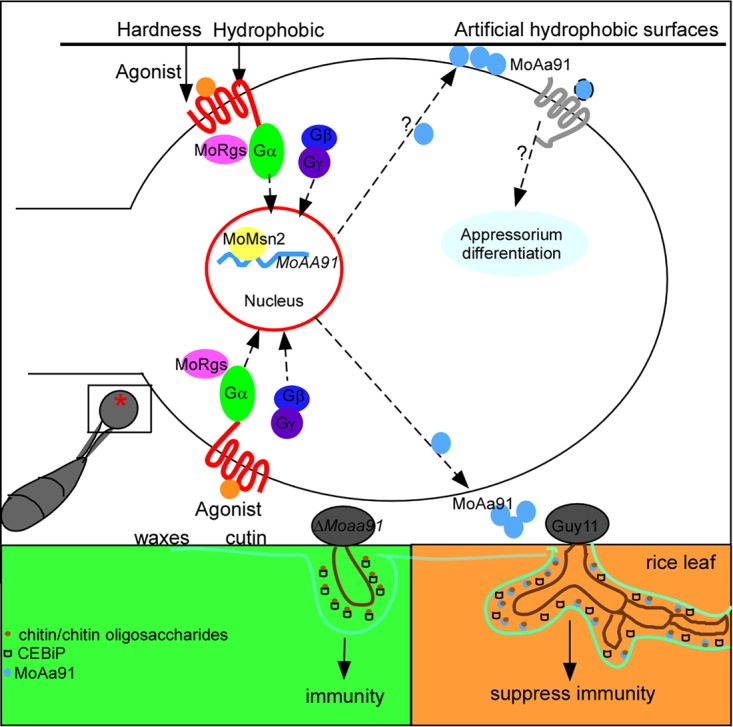
A proposed model depicting MoAa91 functions. The M. oryzae appressorium develops on the artificial inductive surface and rice leaf. The fungus senses the surface by membrane receptor to integrate it into intracellular signaling, resulting in downregulated expression of transcriptional factor *MoMSN2*, which induces the expression of *MoAA91*. MoAa91 is secreted extracellularly and then recognized by the unknown receptors of the fungus, thereby regulating appressorium formation. While on the rice leaf, M. oryzae recognizes surface signals (cutin or wax) and bypasses MoAa91 to form normal appressoria. The wild-type strain Guy11-secreted MoAa91 protein likely acts as an effector to suppress host immunity during infection by competing with CEBiP for chitin binding and thus contributes to disease development. During infections by the Δ*Moaa91* mutant, CEBiP binds with chitin to stimulate immune responses. The upper part refers to presence on the artificial inductive surface, and the lower part refers to presence on rice leaves.

To understand the mechanism by which MoAa91 subverts the host-derived immunity, we truncated MoAa91 into a C-terminal chitin-binding domain and an N-terminal glycoside hydrolase 61 domain. Analysis revealed that the chitin-binding domain was able to complement the defects in virulence of the Δ*Moaa91* mutant sufficiently. Therefore, we propose that MoAa91 suppression of the host defense depends on the chitin-binding activity. Chitin oligosaccharide released from the cell wall of M. oryzae was sensed by CEBiP in rice and triggered chitin-mediated immunity. We speculated that MoAa91 bound with chitin or fungal polysaccharides to block the immune recognition by CEBiP. The secreted protein LysM protein 1 (Slp1) functions in the apoplast space by binding of chitin, thereby preventing the initiation of pattern-triggered immunity by CEBiP (18). Also, Ustilago maydis secretes Rsp3 protein, which binds to fungal hyphae for protection against maize alpha-fetoprotein (AFP) proteins and interacts with membrane-bound receptor kinases containing the DUF26 domain to block signaling and prevent maize immune responses (56). In our study, we found that MoAa91 showed a high affinity in chitin binding in competition with CEBiP, indicating that M. oryzae might secret MoAa91 to compete with CEBiP. As MoAa91 and MoSlp1 showed similar functions in chitin binding, we performed a qRT-PCR assay to reveal the relationship between MoAa91 and MoSlp1 during infection. The results showed that both MoAa91 and MoSlp1 were expressed in the early stage of infection (8 to 24 hpi) and that the expression level of MoAa91 reached a high value at 8 hpi in comparison to that seen at 24 hpi with MoSlp1 (see Fig. S7F and G in the supplemental material). On the basis of these results, we speculated that MoAa91 and MoSlp1 might collaboratively function over the duration of the early infection to subvert host-derived immunity through chitin binding. We have proposed a working model of the regulatory mechanism underlying the function of MoAa91 during appressorium formation and plant infection in M. oryzae (Fig. 11).

10.1128/mBio.03304-19.7 FIG S7 The expression of proteins and qRT analysis of *MoAA91* and *MoSLP1* during the early stage of infection. (A to D) Proteins of Coomassie blue staining. (A) Expression of MoMsn2-His fusion protein. (B) Expression of MoAa91-GST and MoAa91^DTB^-GST fusion protein. (C) Expression of MoAa91^N^ (three transformants) and MoAa91^C^-GST fusion protein. (D) Expression of fusion protein CEBiP-His (two transformants). (E) The standard curve of the reducing sugar determined by DNS assay. The *x*-axis data represent the absorbance at 540 nm, the *y*-axis data refer to the glucose content. The data represent means ± standard errors obtained from the three experimental replicates. (F and G) qRT analysis of *MoAA91* and *MoSLP1* during the early stage of infection. Expression levels of *MoAA91* (F) and *MoSLP1* (G) were analyzed by qRT-PCR (HiScript II Q RT SuperMix; Vazyme Biotech Co., Nanjing, China) during different infection-related stages. RNA samples were extracted from rice plants infected by wild-type Guy11 in mycelium and conidia at 8, 16, 24, 36, 48, and 72 hpi. Error bars represent the standard deviations. Download FIG S7, TIF file, 0.6 MB. Copyright © 2020 Li et al. 2020 Li et al. This content is distributed under the terms of the Creative Commons Attribution 4.0 International license.

## MATERIALS AND METHODS

### Fungal strains and RNA samples.

M. oryzae strain Guy11 was used as the wild type and Δ*Morgs1*, Δ*Morgs3*, Δ*Morgs4*, Δ*Morgs7*, and Δ*Momsn2* mutants as the mutant strains in this study (21, 29). Rice plants (cv. CO-39; 2 weeks old) were inoculated (by standard spray inoculation) with conidial suspensions (3 × 10^6^ spores/ml, in a 0.2% [wt/vol] gelatin solution) of the Guy11, Δ*Morgs1*, Δ*Morgs3*, Δ*Morgs4*, or Δ*Morgs7* strain. The inoculated plants were placed in a sealed plastic box in the dark for 24 h at 28°C, and leaf tissues were collected at 0, 1, 4, and 24 h postinoculation (hpi). Recovered samples were immediately frozen in liquid nitrogen, lyophilized, and stored at –80°C until needed.

### RNA isolation, library construction, and sequencing.

Total RNA was extracted from samples using an Invitrogen kit as described previously (42). The RNA integrity numbers (RIN) of all samples were above 6.8, and 60 cDNA libraries were constructed, and Illumina sequencing was completed by Beijing Genomics Institute (BGI; Shenzhen, China) (57).

Isolation of poly(A) mRNA from total RNA and construction of cDNA libraries were performed according to methods described previously by Dong et al. (26). Finally, the cDNA libraries were loaded onto the flow cell channels of an Illumina HiSeq 4000 platform for paired-end 151-bp-by-2 sequencing at the Beijing Genomics Institute (BGI; Shenzhen, China) (57).

After discarding low-quality raw reads (retaining the connectors and discarding the reads whose content had more than 5% unknown bases), the clean reads from each library were assembled for M. oryzae. Gene expression levels were measured in the RNA-Seq analysis and expressed as the number of exon model reads per kilobase per million (RPKM) (58). Expression levels of differentially expressed genes and the corresponding *P* values were determined using the DEG fold change (log_2_ ratio) values estimated according to the normalized gene expression level in each sample. We used the absolute value of a log_2_ ratio of >1, *P* values of <0.001, and false-discovery-rate (*q*) values of <0.001 as the thresholds to judge differentially expressed genes.

### Secreted-protein-encoding gene predictions.

Proteins that contained signal peptide cleavage sites but not transmembrane helices were selected as putative secreted proteins. Signal peptide cleavage sites were predicted using SignalP 3.0 (http://www.cbs.dtu.dk/services/SignalP-3.0/). TMHMM 2.0 (http://www.cbs.dtu.dk/services/TMHMM-2.0/) was used to predict transmembrane helices.

### Quantitative RT-PCR assay.

Quantitative real-time RT-PCR (qRT-PCR) assays were performed as previously described (26, 42). RNA-Seq expression profiles were validated by qRT-PCR. Primer pairs used as described in this section are listed in Table S1G in the supplemental material.

### Gene disruption and complementation.

To generate *MoAA91* gene replacement vector pCX62, approximately 1-kb upstream and 1-kb downstream fragments were amplified with primer pairs (Table S1G). The resulting PCR products were ligated to the hygromycin resistance cassette released from pCX62, as previously described (26). Putative mutants were screened by PCR and confirmed by Southern blotting. The complement assay was performed according to our previously established methods (26, 29).

### EMSA.

The full-length DNA of the *MoAA91* promoter was amplified from Guy11 genomic DNA using primers *MoAA91*-Pro-F/*MoAA91*-Pro-R (Table S1G). The DNA fragment from the *MoAA91* promoter was end labeled with Alex 660 by PCR amplification using the 5′-Alex 660-labeled primer. The MoMsn2 protein was expressed and purified from Escherichia coli strain BL21 by the use of the pGEX4T-2 construct containing an N-terminal β-glucuronidase (GST) tag coding sequence. The purified protein was mixed with Alex 660-labeled DNA, incubated for 20 min at 25°C in binding buffer, and separated by agarose gel electrophoresis. Gels were visualized directly using a Li-COR (Lincoln, NE, USA) Odyssey scanner with excitation at 700 nm (41).

### Pathogenicity, plant infection, and rice-sheath penetration assays; appressorium formation assay; and appressorium turgor determination.

Conidia were harvested from 10-day-old straw decoction and corn (SDC) agar cultures, were filtered through three layers of lens paper, and were resuspended to a concentration of 5 × 10^4^ spores/ml in a 0.2% (wt/vol) gelatin solution. For the leaf assay, more than 20 leaves from 2-week-old seedlings of rice (Oryza sativa cv. CO39) were used for spray inoculation. Spray inoculation and pathogenicity assays were performed according to methods previously described by Zhang et al. (21). At the same time, more than 20 leaves from 3-week-old seedlings of rice (Oryza sativa cv. CO39) were used for injection inoculation. A 1-ml syringe was used with a concentration of 5 × 10^4^ spores/ml in a 0.2% (wt/vol) gelatin solution for injection of the spore suspension into the cavity of each of the rice sheaths, as the standard method of preparation of infected leaf-sheath material involves filling the hollow space in the center of the sheath with a spore suspension.

For infection assays performed with rice tissues, 3-week-old rice cultivar CO39 was inoculated with 100 μl conidial suspension (1 × 10^5^ spores/ml in a 0.2% [wt/vol] gelatin solution) on the inner leaf-sheath cuticle cells followed by incubation under humid conditions at 28°C. The leaf sheaths were observed under a Zeiss Axio Observer A1 inverted microscope at 30 hpi. A total of 5 leaf sheaths were used for each strain, and this experiment was performed three times.

For the appressorium formation assay, droplets (30 μl) of the conidial suspension were placed on plastic coverslips (Fisher Scientific, St. Louis, MO, USA) under humid conditions at 28°C (59). The appressorium turgor was determined by cell collapse assay using a 1-to-4 molar concentration of glycerol solution. The percentages of conidium-germinating and conidium-forming appressoria were determined by microscopic examination of at least 100 conidia.

### Cutin monomer, wax, and plant extraction treatments.

Cutin monomer (1,16-hexadecanediol; Sigma-Aldrich, United Kingdom) was eluted at 200 μM. Wax (1-octacosanol; Sigma-Aldrich, United Kingdom) was dissolved to 4 mg/ml in chloroform. And aliquots of 10 μl were dropped onto microscope glass surface (Gold Seal, Portsmouth, NH), as previously described (3, 60). The onion epidermis (0.7 g/5 ml double-distilled water [ddH_2_O]) and rice leaves of 2-week-old seedlings (0.43 g/2 ml ddH_2_O) were ground. After centrifugation, the supernatant was subjected to 100× dilution. Drops of 20-μl conidial suspensions were placed on cutin-coated or wax-coated areas, and then the drops were mixed with 1-μl plant extractions and were placed onto plastic coverslips and then assayed for appressorium formation.

### DAB staining and penetration assay performed with DPI or CAG treatment.

For DAB staining assay, rice tissues infected by M. oryzae strains at 30 hpi were stained with 1 mg/ml DAB (Sigma-Aldrich) solution (pH 3.8) for 8 h and destained with an ethanol/acetic acid solution (ethanol/acetic acid = 98:2 [vol/vol]) for 1 h. For evaluation of the growth of IH in ROS-suppressed rice sheath, 0.5 μM DPI or 0.2 U catalase of Aspergillus niger (CAG; Sigma) dissolved in 10 mM (NH4)_2_SO_4_ was mixed with the conidial suspensions (1 × 10^5^ spores/ml) to suppress the stress imposed by ROS. The details of the methods used were as previously described (39–42). All the samples were observed under a Zeiss Axio Observer A1 inverted microscope (40×).

### *In vitro* protein purification assays.

To construct the plasmids corresponding to *GST*-*MoAA91* and *GST*-*MoAA91*^DTB^, *His*-*MoMSN2*, and *His*-*CEBiP*, full-length cDNA of *MoAA91* lacking the signal peptide was amplified and inserted into the vector pGEX4T-2, full-length cDNA of *MoMSN2* was amplified and inserted into the vector pET-32A, and full-length cDNA of *CEBiP* lacking the signal peptide and the transmembrane domain was amplified and inserted into the vector pET-32A, respectively. These plasmids were then expressed in E. coli strain BL21, and bacterial cells were collected and treated with lysis buffer (10 mM Tris-HCl [pH 7.5], 150 mM NaCl, 0.5 mM EDTA, 0.5% Triton X-100). To confirm the expression of the GST or His fusion proteins, bacterial lysates were separated by the use of SDS-PAGE gel followed by Coomassie blue staining (see Fig. S7A to D in the supplemental material). In the assay used for purification of protein (His-MoMsn2/CEBiP and GST-MoAa91/MoAa91^DTB^), bacterial lysate containing His-MoMsn2/CEBiP protein was incubated with 30 μl nickel-nitrilotriacetic acid (Ni-NTA) agarose beads (Invitrogen, Shanghai, China) and bacterial lysate containing GST-MoAa91/MoAa91^DTB^ protein was incubated with GST beads for 1 h at 4°C. The beads were then washed five times, and the elution was reduced using glutathione (Abmart, Shanghai, China).

### Synergistic cellulase hydrolysis with MoAa91.

For the hydrolysis of cellulose, 1% (wt/vol) Avicel PH-101 (Sigma-Aldrich, United Kingdom) was incubated with 1 mg of MoAa91 or MoAa91^DTB^/g Avicel in the presence or absence of Celluclast (Sigma-Aldrich, United Kingdom) in 50 mM sodium acetate buffer (pH 5.0) at 50°C for 48 h. Sodium azide (NaN_3_) was added at 0.02% (wt/vol) to all the synergistic reaction mixtures as an antibiotic or reducing agent. To terminate the enzyme reaction, the reaction mixture was boiled at 95°C for 5 min and was then subjected to centrifugation at 12,000 × *g* for 5 min. Finally, the amount of reducing sugar present in the supernatant was quantified by 3,5-dinitrosalicylic acid (DNS) assay at 540 nm using d-glucose as the standard (61) (Fig. S7E). The extent of synergism was equal to the yield of reducing sugar from the hydrolysis of the MoAa91 or *ΔMoaa91* mutant with the cellulase.

### Affinity precipitation of MoAa91 with polysaccharides.

The affinity of MoAa91 for various polysaccharides was investigated by incubating 50 mg/ml of MoAa91 with 5 mg of chitin beads (New England Biolabs), crab shell chitin, xylan, or cellulose (Avicel; Sigma-Aldrich) as described previously (18, 62). Protein and the polysaccharide were incubated at 4°C on a rocking platform in a final volume of 1 ml of water. After 16 h, the insoluble pellet fraction was centrifuged (5 min, 13,000 × *g*), and the supernatant was collected. The insoluble fraction was pelleted and rinsed a further three times in distilled sterile water to remove unbound protein. Both the supernatant and the pelleted fractions were then boiled in 200 ml of 1% SDS solution before being examined by SDS-PAGE and Western blotting.

### Data availability.

The gene identity (ID) in this paper can be found in the FungiDB (https://fungidb.org/fungidb/). And the accession number for the transcriptome used in this study is GSE128219.
