# Supplementary figures and images for: Magnaporthe oryzae Auxiliary Activity Protein MoAa91 Functions as Chitin-Binding Protein To Induce Appressorium Formation on Artificial Inductive Surfaces and Suppress Plant Immunity
Source: mBio. 2020 Mar 24;11(2):e03304-19. doi: 10.1128/mBio.03304-19 (PMC7157532; doi:10.1128/mBio.03304-19)

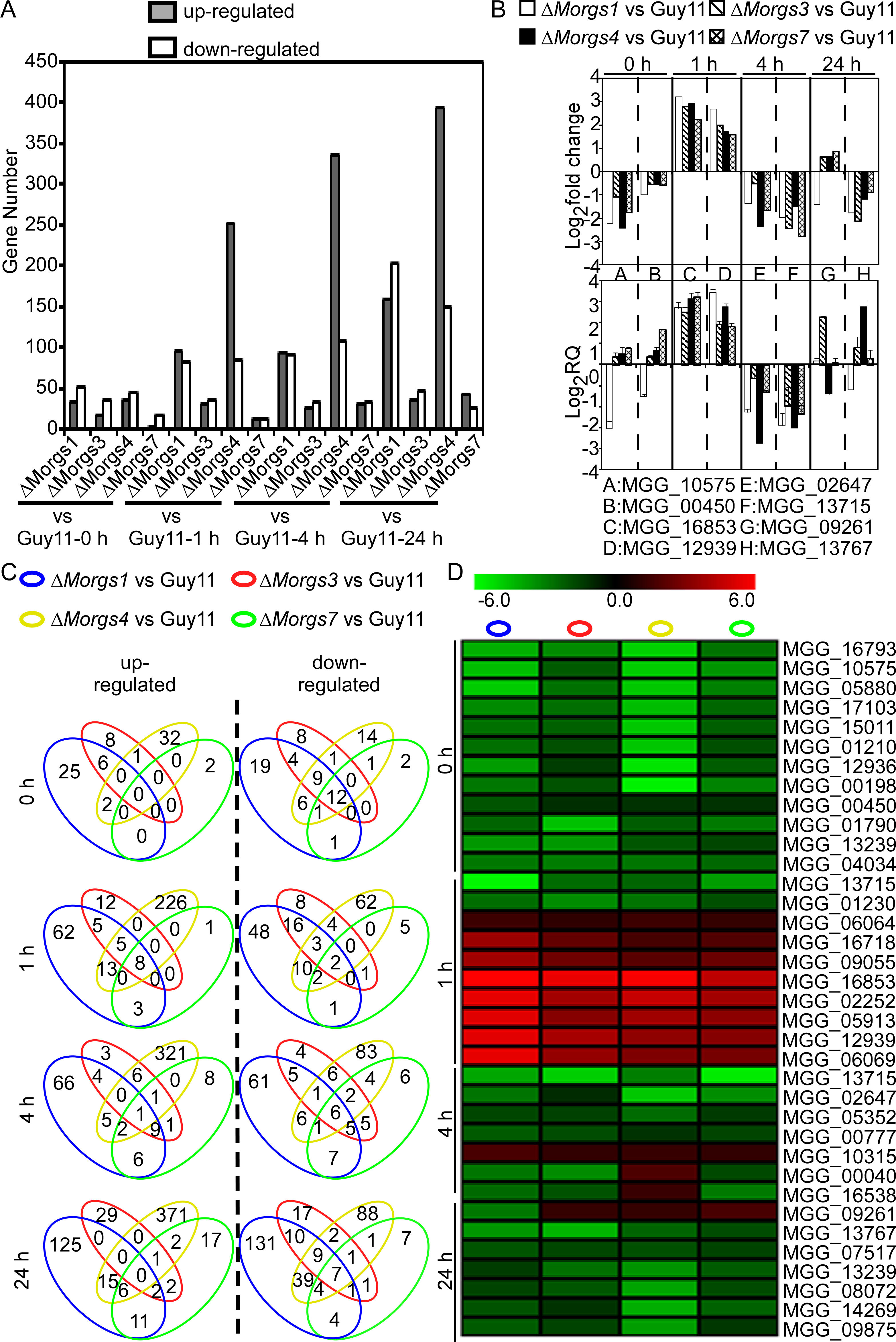

Supplement: FIG S1 [file mBio.03304-19-sf001.tif]

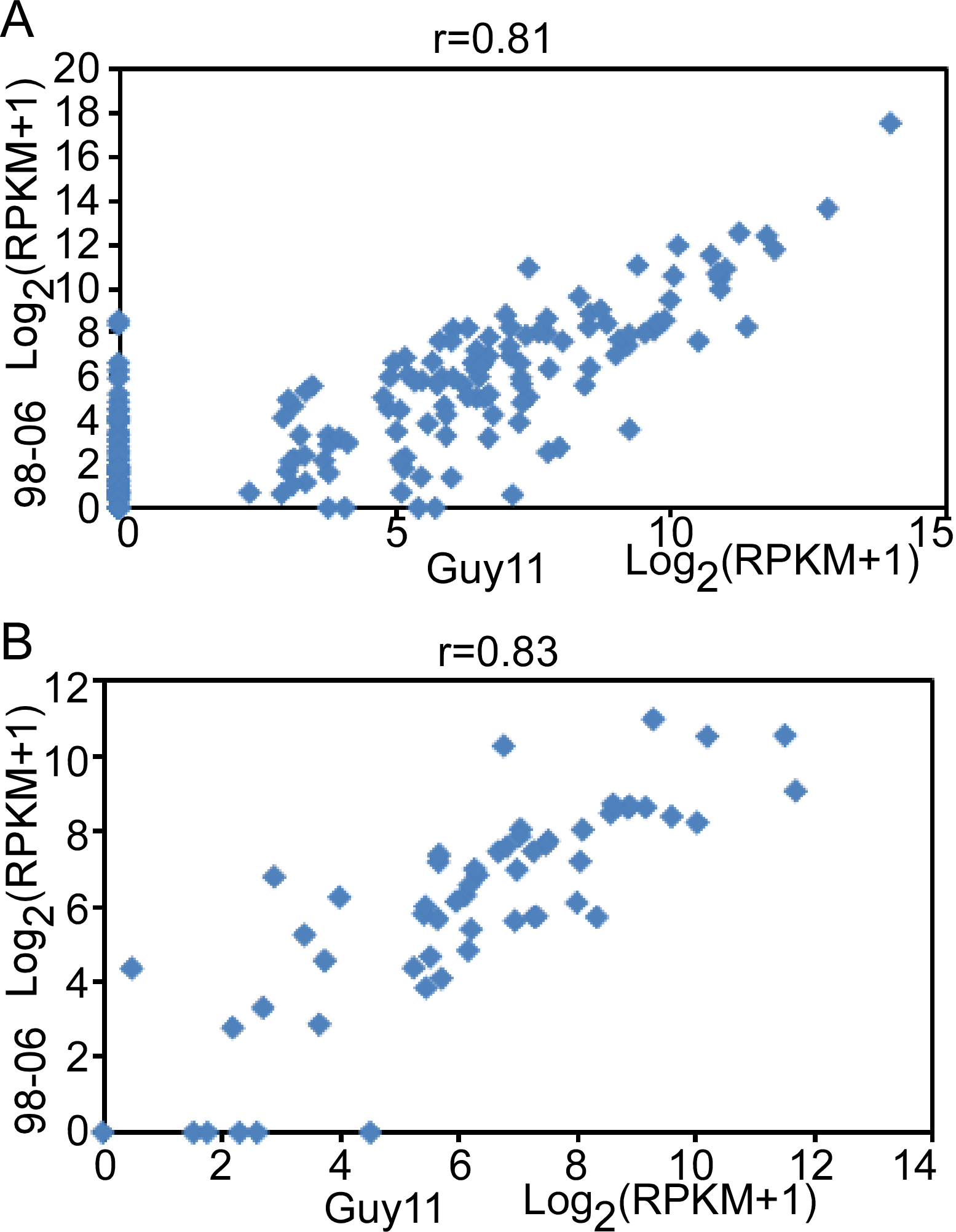

Supplement: FIG S2 [file mBio.03304-19-sf002.tif]

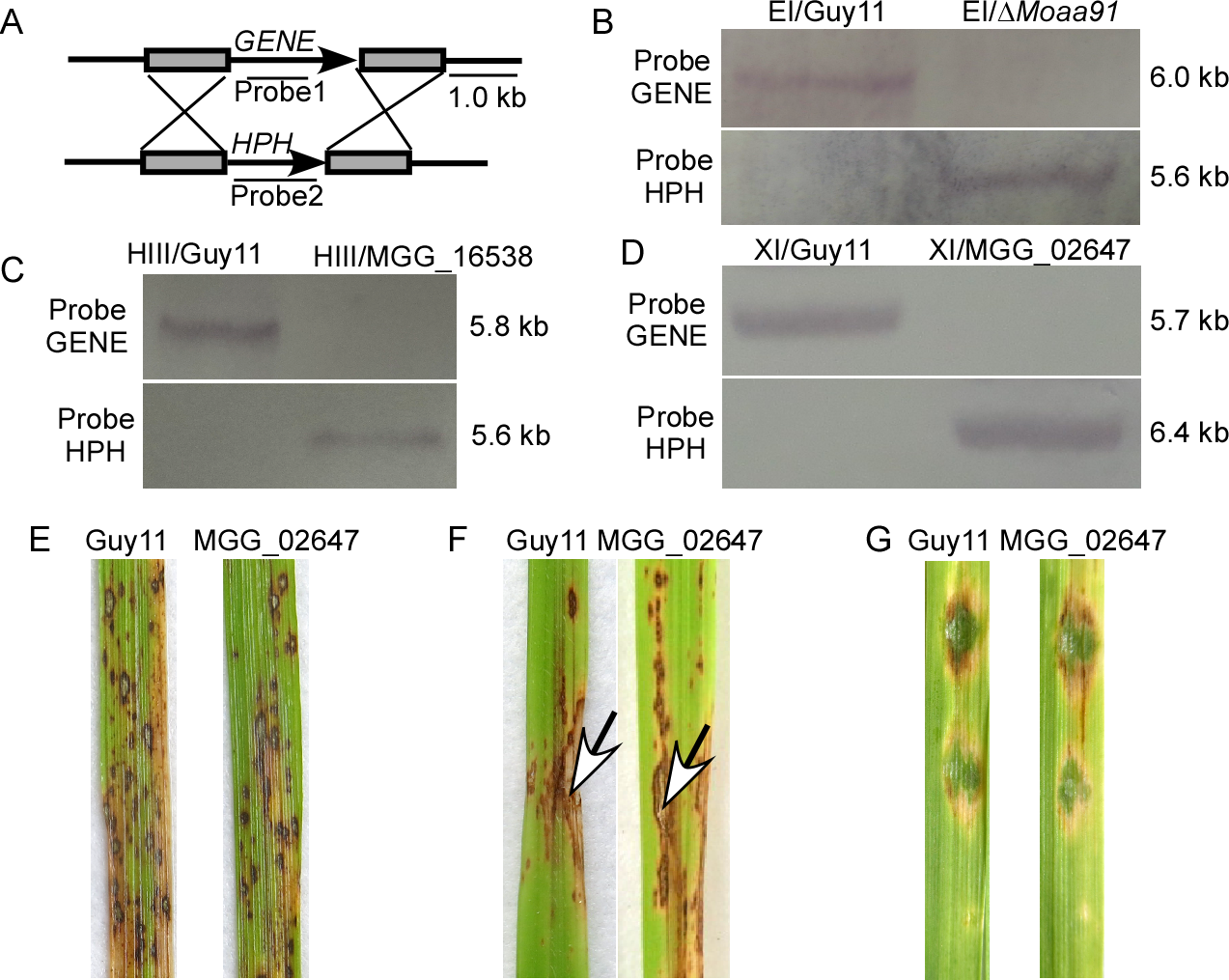

Supplement: FIG S3 [file mBio.03304-19-sf003.tif]

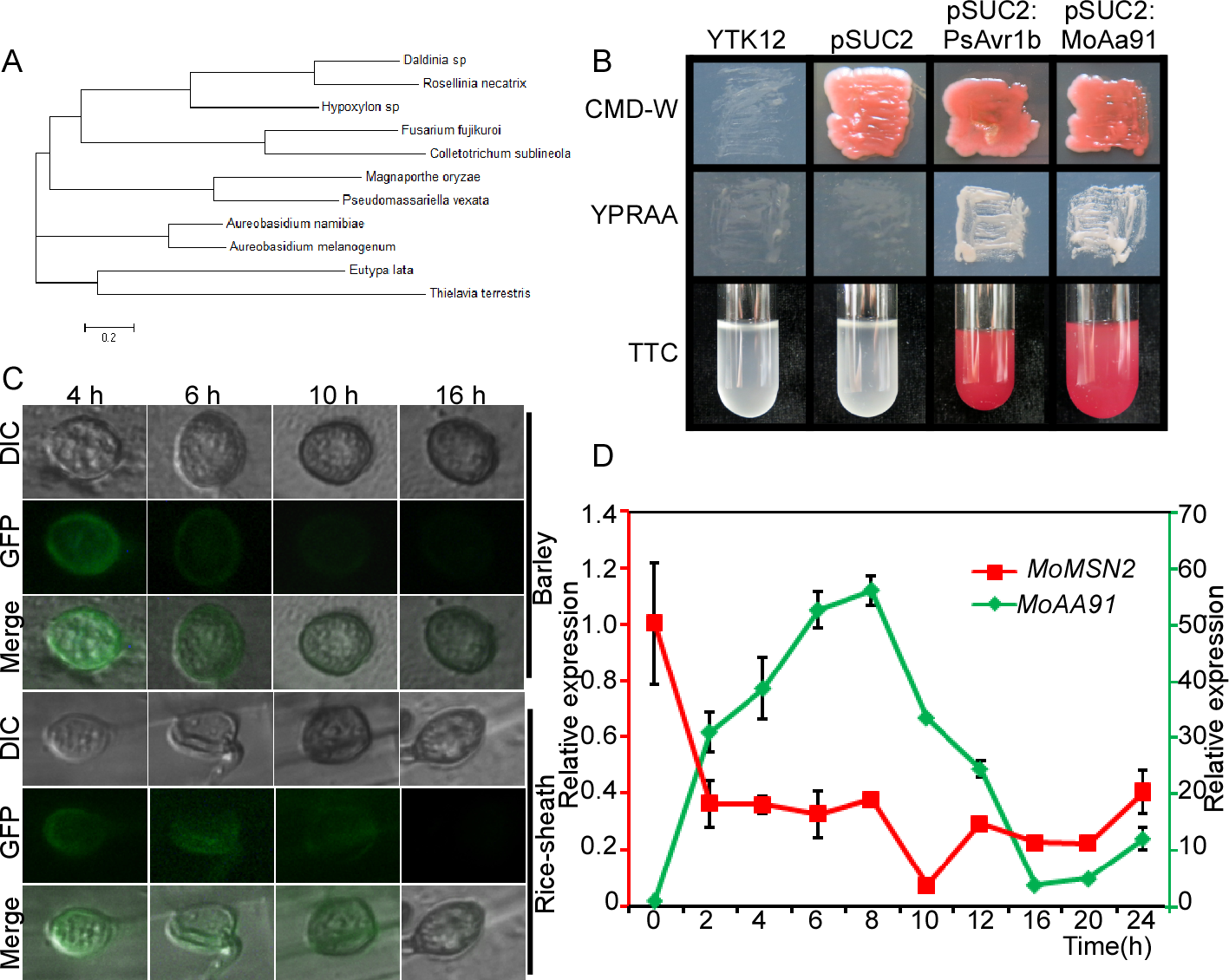

Supplement: FIG S4 [file mBio.03304-19-sf004.tif]

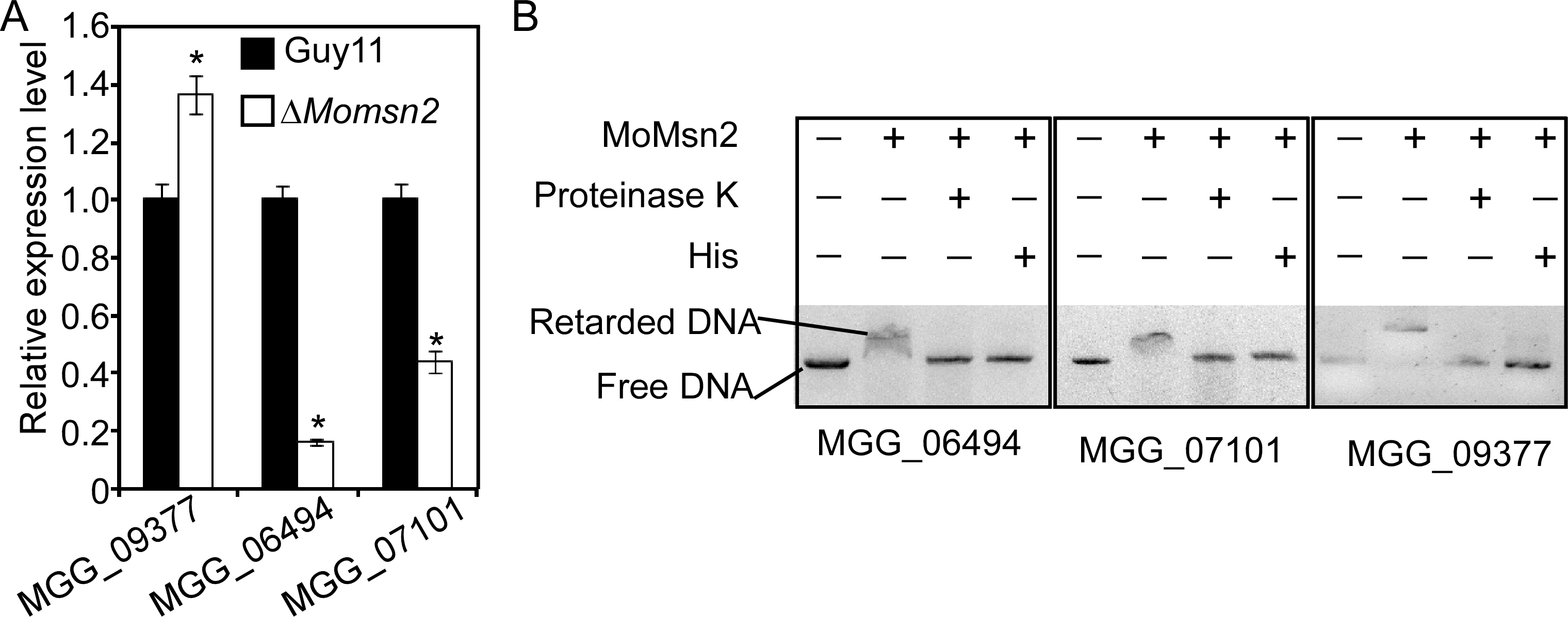

Supplement: FIG S5 [file mBio.03304-19-sf005.tif]

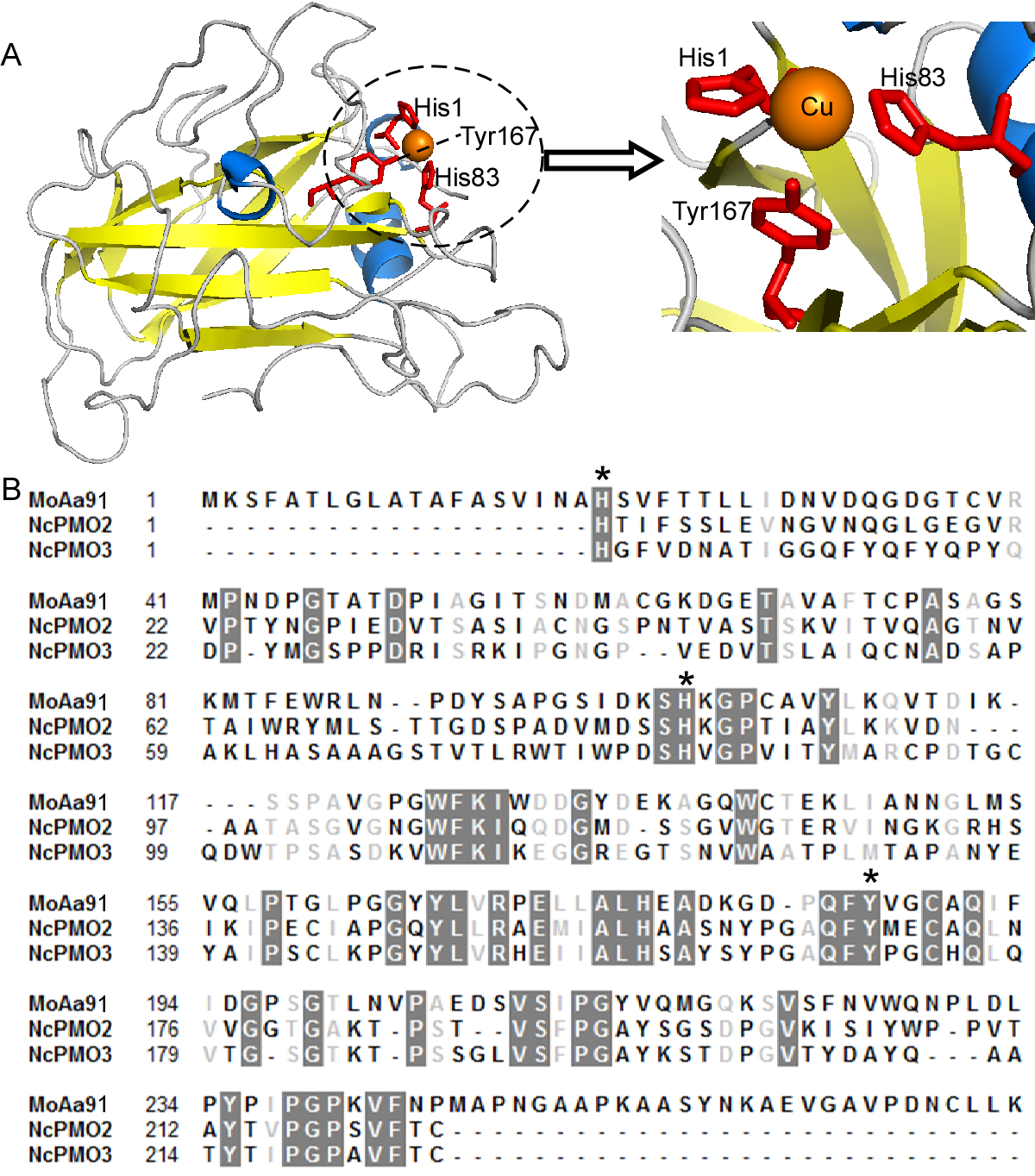

Supplement: FIG S6 [file mBio.03304-19-sf006.tif]

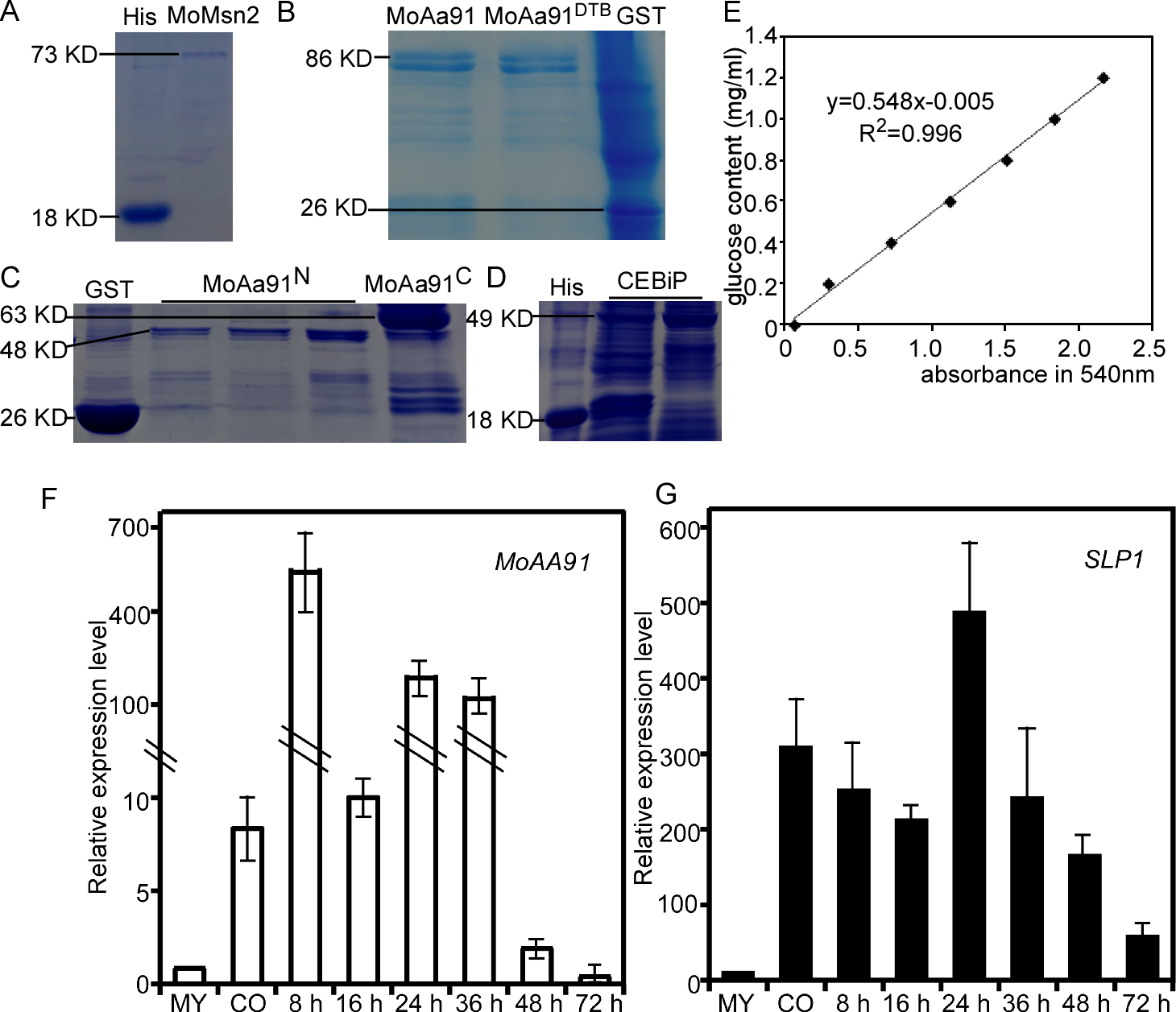

Supplement: FIG S7 [file mBio.03304-19-sf007.tif]
